# Increased expression of individual genes in whole blood is associated with late-stage lung cancer at and close to diagnosis

**DOI:** 10.1038/s41598-023-48216-z

**Published:** 2023-11-25

**Authors:** Ilona Urbarova, Anne Heidi Skogholt, Yi-Qian Sun, Xiao-Mei Mai, Bjørn Henning Grønberg, Torkjel Manning Sandanger, Pål Sætrom, Therese Haugdahl Nøst

**Affiliations:** 1 Department of Community Medicine, Faculty of Health Sciences, UiT The Arctic University of Norway, Tromsø, Norway; 2 Department of Public Health and Nursing, Norwegian University of Science and Technology, Trondheim, Norway; 3 Department of Clinical and Molecular Medicine, NTNU, Norwegian University of Science and Technology, Trondheim, Norway; 4 Department of Pathology, Clinic of Laboratory Medicine, St. Olavs Hospital, Trondheim University Hospital, Trondheim, Norway; 5 Center for Oral Health Services and Research Mid-Norway (TkMidt), Trondheim, Norway; 6 Department of Oncology, St. Olavs Hospital, Trondheim University Hospital, Trondheim, Norway; 7 Bioinformatics Core Facility, Norwegian University of Science and Technology, Trondheim, Norway

**Keywords:** Lung cancer, Lung cancer, Epidemiology, Cancer epidemiology, Diagnostic markers, Prognostic markers

## Abstract

Lung cancer (LC) mortality rates are still increasing globally. As survival is linked to stage, there is a need to identify markers for earlier LC diagnosis and individualized treatment. The whole blood transcriptome of LC patients represents a source of potential LC biomarkers. We compared expression of > 60,000 genes in whole blood specimens taken from LC cases at diagnosis (n = 128) and controls (n = 62) using genome-wide RNA sequencing, and identified 14 candidate genes associated with LC. High expression of *ANXA3, ARG1* and *HP* was strongly associated with lower survival in late-stage LC cases (hazard ratios (HRs) = 2.81, 2.16 and 2.54, respectively). We validated these markers in two independent population-based studies with pre-diagnostic whole blood specimens taken up to eight years prior to LC diagnosis (n = 163 cases, 184 matched controls). *ANXA3* and *ARG1* expression was strongly associated with LC in these specimens, especially with late-stage LC within two years of diagnosis (odds ratios (ORs) = 3.47 and 5.00, respectively). Additionally, blood CD4 T cells, NK cells and neutrophils were associated with LC at diagnosis and improved LC discriminative ability beyond candidate genes. Our results indicate that in whole blood, increased expression levels of *ANXA3, ARG1* and *HP* are diagnostic and prognostic markers of late-stage LC.

## Introduction

Lung cancer (LC) is the deadliest cancer, with an estimated 1.8 million deaths in 2020 worldwide^1^, causing more deaths than breast, prostate, colorectal, and brain cancers combined. Although a decreasing trend in LC mortality rates is observed mainly in Europe, North America and several other high-income countries in the world^2,3^, LC mortality rates are still increasing globally, with strong geographical differences linked to smoking patterns and air quality^1^. The overall five-year survival of patients with all types of LC is less than 20%, although grouped by stage, survival varies from 50% for early- to only 2% for late-stage – owing mostly to distant metastasis^4,5^. Further, survival differs between the two main histological subtypes of LC. Non-small cell LC (NSCLC) with the dominating entities adenocarcinomas (AD) and squamous cell carcinomas (SQ) and small cell lung cancer (SCLC) have overall five-year survival rates of around 25% and 7%, respectively^4^. However, these differences reflect primarily the rapidly developing nature of SCLC, which is typically diagnosed at a distant metastasis stage^6^.

As survival is linked to stage, there is a need to identify markers for earlier LC diagnosis and individualized treatment. To increase diagnostic and prognostic precision, several DNA, RNA and protein biomarker candidates have been identified, mainly based on LC tissue biopsies^7–9^. However, their diagnostic ability and clinical utility are variable, and their implementation is challenging. Multi-gene expression biomarker panels have successfully led to more accurate classification to improve prognosis in breast, prostate and colon cancer in clinical settings^10–14^. However, identification of similar biomarkers in LC is more complicated due to large expression-based heterogeneity independent of LC histology^15^, particularly for solid tumour specimens^16,17^. In contrast to tissue biopsies, gene expression in blood is non-invasive, but can represent a surrogate measurement of tissue-specific gene expression and potentially also aid in disease predictions^18^. Specifically, gene expression profiling of whole blood specimens, separated blood cells, or cell-free RNA in blood from LC patients^15,19–22^ suggests that LC development and progression can be detected as systemic alterations in whole blood. However, whole blood gene expression studies should also consider that expression profiles of specimens could be influenced by immune cell composition^23^. Indeed, elevated blood neutrophil-to-lymphocyte ratio (NLR) has been shown to have a prognostic value in patients in several studies, both at^24,25^ and close to LC diagnosis^26^, suggesting that gene expression changes observed in whole blood might only reflect changes in immune cell composition. Whereas many studies have investigated gene expression differences between cases and controls in blood specimens sampled at LC diagnosis^15,19–21,27–29^, investigations in pre-diagnostic blood specimens are rare^30,31^. Consequently, it is unclear whether diagnostic whole blood gene expression markers of LC are predictive of LC in pre-diagnostic blood specimens.

In this work, we used genome-wide expression data from three studies, one at diagnosis and two prospective studies. We identified LC candidate markers using whole blood specimens taken at diagnosis in confirmed LC cases compared to individuals with suspected but confirmed negative LC evaluation, which resembles a screening situation. Further, we evaluated case–control differences and trends with time between specimen collection and LC diagnosis for the LC candidate markers in the two prospective studies.

## Methods

### Study sample

Analyses in this work were based on whole blood specimens, questionnaires and registry-based data from three different studies; one diagnostic, hospital-based study: the Norwegian Lung Cancer Biobank (NLCB), and two prospective, population-based studies: the Norwegian Women and Cancer Study (NOWAC) and the Trøndelag Health Study 3 (HUNT3). The diagnostic study includes blood specimens collected during a detailed clinical evaluation of lung cancer (LC) and comprises individuals with confirmed positive LC diagnosis (LC cases) and individuals who underwent diagnostic workup on suspicion of LC, but who were confirmed not to have LC (suspected LC cases). The suspicion of LC was mainly based on findings on imaging, mainly CT scans. The suspected LC cases were included as a control group in this work and considered “false positives” (hereafter referred to as FalsePos), as this scenario resembles a realistic LC screening situation compared to inclusion of healthy individuals.

In the prospective studies, only blood specimens collected prior to LC diagnosis (cases) and specimens from matching individuals never diagnosed with cancer (controls) were included in the study; these were identified using linkages to national cancer registries in Norway. Cases included specimens taken up to eight years prior to LC diagnosis. The matching criteria between cases and controls slightly differed in the two prospective studies (Supplementary Methods). In all studies, histological subtypes considered were non-small cell LC (NSCLC) including adenocarcinoma (AD), squamous cell carcinoma (SQ) and a group of other NSCLCs; and small cell LC (SCLC).

### Definition of LC cases

In all three studies, patients with LC were defined as persons registered with International Classification of Diseases (ICD-10) topography codes C33–C34. We classified LC cases as early-, middle-, and late-stage based on information from medical journals for data in the NLCB study (TNM status), and the national cancer registry for data in the NOWAC and HUNT3 studies (classification by the registry, see Supplementary Table S1). The aim was to construct staging information that could be harmonized across cohorts and to describe early-stage cases as a group with local disease, middle-stage cases as a group with a regional disease and regional spread, and late-stage cases as a group with advanced and systemic disease that had spread to the whole body.

### Candidate gene identification in the diagnostic study

The whole analysis workflow is visualized in Supplementary Fig. S1. RNA-seq data in the diagnostic study (NLCB) were generated and processed as described in the Supplementary Methods. Differentially expressed (DE) genes were identified using the Bioconductor package *limma* v3.46.0 combined with *voom* transformation^32,33^. Expressed genes were represented by log_2_ reads per million (RPM) values in limma models and contrasts were defined by stage and histological subtype information. All models used to identify candidate genes included case status (LC case or FalsePos), stage status (early-, middle- or late-stage LC), histological subtype (AD, SQ, Other NSCLCs, SCLC), smoking status (never/ever), age (scaled) and sex in addition to technical variation (Supplementary Fig. S2);* p*-values were false discovery rate (FDR)-adjusted using Benjamini–Hochberg method^34^. We included several subcomparisons to identify DE genes: (i) cases versus FalsePos, (ii) AD versus SQ, (iii) AD versus Other NSCLC, (iv) SQ versus Other NSCLC, (v) SCLC versus NSCLC, (vi) late-stage cases versus FalsePos, (vii) NSCLC versus FalsePos, and (viii) early-stage versus late-stage cases. Identified significantly DE genes from these subcomparisons were further investigated using pathway enrichment analyses (R package *ReactomePA* v1.34.0)^35^ and the top five and ten enriched pathways for up- and downregulated genes, respectively, were visualized in separate plots using *dotplot* function from *enrichplot* R package v1.10.2^36^.

We applied a stringent filtering approach that filtered several lowly expressed genes with high absolute log_2_ fold change values (logFC expression-dependent filtering) from the significantly DE genes found in our analyses. This was done to find more robust candidate markers of LC disease^37,38^ that could be associated with LC also in other cohorts. Specifically, we fitted a locally estimated smoothing (loess) regression curve on the average gene expression and absolute logFC for all significantly DE genes detected in any of the case–control subcomparisons, and used the curve to define a stricter cut-off for the logFC expression-dependent filtering (Supplementary Fig. S3). This approach filtered out genes with low average expression but high absolute logFC values found as significantly DE in our analysis due to heteroscedasticity in RNA sequencing (RNA-seq) data, compared to the classical filtering approach that considers a logFC cut-off only. Candidate genes in the diagnostic study were defined using the following criteria; (i) absolute fold change (FC) values larger than 1 for tests with significant DE genes, i.e., between all LC cases, NSCLC cases or late-stage LC cases compared to FalsePos; (ii) FDR-adjusted* p*-value lower than 0.05; and (iii) absolute logFC values above the expression-dependent loess curve (Supplementary Fig. S3). Correlations in expression of the candidate genes were computed using log_2_RPM values and the *corrplot* R package v0.92^39^ (Pearson correlation coefficients). Survival models used vital status (follow-up until August 2018) including cancer stage (stratified into early-, middle- and late-stage), age (scaled), sex, and smoking status (never/ever) in the *Surv* function in the R package *survival* v3.2–13^40^.* P*-values were adjusted using the Benjamini–Hochberg correction. The ability of candidate genes to discriminate between case/FalsePos in the diagnostic study was investigated using logistic regression models (*glm* function, R package *stats* v4.0.5) and receiver operating characteristic (ROC) curves (*roc* function, R package *pROC* v1.18.0)^41^.

### Candidate gene evaluation in the prospective studies

RNA-seq and microarray data in the prospective studies (NOWAC and HUNT3) were generated and processed as described in Supplementary Methods. We investigated the expression of candidate genes in the prospective studies focusing on the comparisons between: (i) all LC cases versus all controls, (ii) late-stage LC cases versus all controls, and (iii) NSCLC cases versus all controls, as we identified significantly DE genes only in these comparisons in the diagnostic study. Data in the prospective studies were pre-processed as described in Supplementary Methods and analysed as one combined dataset. For the case–control comparisons, we used logistic mixed-effects models (*glmer* function, R package *lme4* v1.1–28)^42^ for joint analyses allowing for inclusion of separate studies as random effect. All individuals from the prospective studies were included in the same model and adjusted for age (scaled), sex, smoking variable (seven categories based on both smoking status [never/current/former] and pack-years; see below for details) and study as random effect. For evaluation of variation in gene expression from blood specimen collection to LC diagnosis, we used mixed-effects models (*lmer* function, R package *lmerTest* v3.1–3)^43^ in combination with generalized additive models (*gam* function, R package *gam* v1.20)^44^. The lmer models included expression values of the candidate genes for the prospective LC cases, adjusted for age (scaled), sex and smoking variable in addition to including study as random effect. Residuals from the lmer models served as input into the gam models. Resulting plots showed cubic regression splines for time in years between blood sampling and LC diagnosis for all cases in both prospective studies. Based on these time to diagnosis plots for two of the identified candidate genes, we restricted the case–control comparisons for these two genes to only those LC cases diagnosed within two years of blood sample collection. The ability of candidate genes to discriminate between case/control status in the prospective studies was investigated similarly as in the diagnostic study.

### Smoking adjustments in the diagnostic and prospective studies

Smoking adjustments used in the models differed between the diagnostic (NLCB) and the prospective studies (NOWAC and HUNT3). Smoking information for LC patients in the diagnostic study could be biased, as it was directly retrieved from individual patients’ medical journals. The smoking information used in the diagnostic study was therefore smoking status with only two categories (never/ever smokers), which is considered the most reliable information in this study.

In the prospective studies, a smoking variable with seven categories was constructed from questionnaires and was based on both smoking status (never/current/former) and pack-years (pyrs) information using the following criteria; (1) never smokers, (2) former smokers ≤ 10.0 pyrs, (3) former smokers 10.1–20.0 pyrs, (4) former smokers ≥ 20.1 pyrs, (5) current smokers ≤ 10.0 pyrs, (6) current smokers 10.1–20.0 pyrs, and (7) current smokers ≥ 20.1 pyrs.

### Blood cell type estimates

There were no available blood counts in these studies. Therefore, we estimated blood cell type proportions from blood DNA methylation (DNAm) data generated for 178 individuals (n = 126 cases, 52 FalsePos) from the diagnostic study included in this project using EpiDISH algorithm^45^, with non-constrained reference-based approach based on robust partial correlations (RPC) and using centDHSbloodDMC.m reference matrix^45,46^. We estimated blood cell type proportions also from gene expression data (the RNA-seq data generated in the NLCB study) using CIBERSORT algorithm^47^. We ran CIBERSORT using normalized, non-transformed expression matrix and LM22 reference file, with disabled quantile normalization, as recommended by the authors of CIBERSORT for RNA-seq data. Logistic regression models (*glm* function, R package *stats* v4.0.5) adjusted for sex, age (scaled) and smoking status (never/ever) were used to evaluate association between LC status and blood cell type estimates.

In the prospective studies, we estimated blood cell type proportions similarly as in the diagnostic study using available blood DNAm data^48,49^. The DNAm data were available for all individuals in the NOWAC study, and for all but four individuals in the HUNT3 study (3 controls, 1 case). Logistic regression models (*glm* function, R package *stats* v4.0.5) adjusted for sex, age (scaled) and smoking variable (seven categories) were used to evaluate association between LC status and blood cell type proportions estimated from DNAm or RNA-Seq/microarray data in each of the prospective studies separately.

Further, we included the cell type estimates significantly different between cases and FalsePos in the diagnostic study in the ROC curve analysis to evaluate the performance of our candidate genes in discrimination of LC cases from FalsePos/controls. In the diagnostic study, we used cell type proportions estimated from blood DNAm data for 178 individuals. For the remaining 12 individuals without blood DNAm data available, we adjusted the cell type proportions estimated from RNA-seq data by using the Spearman’s rank correlation coefficient (R = 0.8 for neutrophils, R = 0.66 for CD4 T cells and R = 0.67 for NK cells) obtained using correlation of cell type proportion estimates from EpiDISH versus CIBERSORT for the 178 individuals having available both the DNAm and gene expression data, respectively (Supplementary Fig. S4). In the prospective studies, we used cell type proportions estimated from blood DNAm data for all individuals in the NOWAC study and for all but four individuals in the HUNT3 study. For the remaining four individuals without blood DNAm data available, we estimated blood cell type proportions from gene expression data (the RNA-seq data generated in the HUNT3 study) using CIBERSORT algorithm^47^ and adjusted them using the Spearman’s rank correlation coefficient (R = 0.69 for neutrophils, R = 0.63 for CD4 T cells and R = 0.7 for NK cells) obtained using correlation of blood cell type proportions estimated from EpiDISH versus CIBERSORT for 92 individuals with both the DNAm and gene expression data available, respectively.

### Ethics approval and consent to participate

All participants have given written informed consent to the respective cohorts and the studies have been approved by the respective Regional Committee for Medical and Health Research Ethics in Norway (REK nord 2016/175 for NOWAC, REK sør-øst 2015/78 for HUNT3 and REK midt 2018/638 for NLCB study). The research has been conducted according to the principles stated in the Declaration of Helsinki.

## Results

### Overview of the study participants and the study design

This work used whole blood specimens, questionnaires, and registry data from three different studies, one hospital-based diagnostic study (NLCB) and two prospective studies (NOWAC and HUNT3; Table 1); see Methods section and Supplementary Methods for further description. NOWAC included only women, whereas there were 42% and 53% women in NLCB and HUNT3, respectively. The analysis workflow is visualized in Supplementary Fig. S1. The diagnostic study (NLCB) was used as a discovery set to identify candidate LC markers, which we evaluated for their potential as LC risk predictors in two independent prospective studies (NOWAC and HUNT3).

**Table 1:** Main characteristics of the participants from the different studies.

	Diagnostic study				Prospective studies			
	NLCB				NOWAC		HUNT3	
	Early-stage	Middle-stage	Late-stage	FalsePos	Cases	Controls	Cases	Controls
n =	23	42	63	62	125	126	38	58
Gender								
Women	9	23	22	26	125	126	21	30
Men	14	19	41	36	–	–	17	28
Smoking status (NLCB)								
Never	1	0	5	11				
Ever	22	42	58	51				
Smoking factor variable* (NOWAC, HUNT3)								
Never					13	51	2	29
Former (≤ 10.0 pyrs)					11	25	3	9
Former (10.1–20.0 pyrs)					10	8	1	4
Former (≥ 20.1 pyrs)					13	3	11	5
Current (≤ 10.0 pyrs)					10	7	4	3
Current (10.1–20.0 pyrs)					20	15	3	3
Current (≥ 20.1 pyrs)					48	17	12	4
Unknown							2	1
Stage status								
Early-stage	23	–	–		28		7	
Middle-stage	–	42	–		31		11	
Late-stage	–	–	63		66		18	
NA	–	–	–		–		2	
Histology								
AD	5	10	27	–	63	–	17	–
SQ	10	14	11	–	18	–	6	–
Other NSCLC	8	14	9	–	20	–	8	–
SCLC	0	4**	16	–	24	–	6	–
NA	–	–	–	–	–	–	–	–
Time to diagnosis (Years between specimen collection and LC diagnosis)								
Mean	–	–	–	–	3.87		3.27	–
(Min–Max)					(0.29–7.92)	–	(1.03–5.43)	
Year of diagnosis								
Min	2006	2006	2006	–	2004	–	2009	–
Max	2012	2012	2012	–	2011	–	2013	–
Age at specimen collection								
Mean	68.1	70.8	66.8	63.3	56.7	56.6	69.3	67.7
(Min–Max)	(51.3–82.0)	(49.4–85.2)	(45.5–86.7)	(31.8–85.5)	(48.0–63.0)	(48.0–63.0)	(49.7–91.7)	(47.8–89.0)

*A smoking variable with seven categories combing smoking status (never/ current/ former) with pack-years (pyrs).

**Localized SCLC (stage III).

*NLCB* = *the Norwegian Lung Cancer Biobank, NOWAC* = *the Norwegian Women and Cancer Study, HUNT3* = *the Trøndelag Health Study 3, AD* = *adenocarcinoma, SQ* = *squamous cell carcinoma, Other NSCLC* = *Other non-small cell lung cancer subtypes, SCLC* = *small cell lung cancer, NA* = *not available.*

### Identification of candidate genes at diagnosis

**Figure 1:**
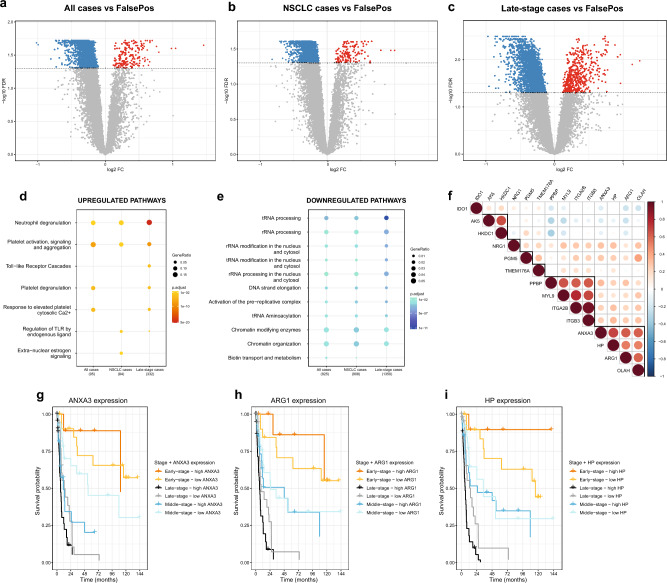
Identification and characteristics of candidate genes in the diagnostic study (NLCB). (**a**, **b**, **c**) Volcano plots for the three case–control comparisons with significantly DE genes: (**a**) all LC, (**b**) NSCLC and (**c**) late-stage LC cases versus FalsePos. Down- and upregulated genes with FDR-adjusted *p*-value (FDR, Benjamini-Hochberg [BH] method) < 0.05 are indicated in blue and red, respectively. (**d**, **e**) Reactome pathway analysis for (**d**) upregulated and (**e**) downregulated genes in the diagnostic study. Only the top (**d**) five and (**e**) ten most significantly enriched pathways (FDR < 0.05) in each comparison are included in this figure. (**f**) Correlation matrix of log_2_ reads per million (log_2_RPM) values of 14 candidate genes from the diagnostic study that were evaluated in the prospective studies (Pearson correlation coefficients). Highly correlated candidates are highlighted with black lines. (**g**, **h**, **i**) Survival curves for three candidate genes with significant* p*-values in Cox models: (**g**) *ANXA3*, (**h**) *ARG1* and (**i**) *HP*; FDR (BH method) for late-stage LC cases were 0.009, 0.03, and 0.007, respectively. High *ANXA3* expression was also significantly related to poor prognosis in middle-stage LC cases (FDR = 0.04). Low or high gene expression was defined as having below or above median log_2_RPM value of this gene (indicated as ‘low’ or ‘high’, respectively).

We generated RNA-seq data from whole blood specimens in the diagnostic study (NLCB) to identify candidate LC markers, comparing gene expression in confirmed LC cases (n = 128) to confirmed negative LC individuals (FalsePos; n = 62). RNA-seq reads were mapped to the human genome reference (hg38), and 60,675 genes were filtered into a final count matrix with 14,014 annotated genes. We compared gene expression for the different LC stage (early-, middle and late-stage) and histological subtypes (AD, SQ, Other NSCLC and SCLC) using adjusted limma models considering eight different subcomparisons (see Methods section for details), and detected significantly DE genes (FDR-adjusted* p*-value < 0.05, Benjamini–Hochberg method) only in the following three comparisons: all LC cases versus FalsePos (Fig. 1a), non-small cell LC (NSCLC) cases versus FalsePos (Fig. 1b) and late-stage LC cases versus FalsePos (Fig. 1c). The most substantial differences were observed for late-stage LC cases compared to FalsePos (Fig. 1c). We detected 2,003, 1,906 and 3,394 DE genes in the three comparisons, respectively, and the majority were downregulated in LC cases (153, 139 and 532 were up- and 1,850, 1,767 and 2,862 were downregulated, respectively; Fig. 1a-c). The DE genes from these three comparisons; i.e., 2,003, 1,906 and 3,394 DE genes, were investigated for enriched pathways using Reactome Pathway Analysis. The top two most significantly (FDR < 0.05) upregulated pathways common in all the three comparisons were ‘neutrophil degranulation’ and ‘platelet activation and degranulation, signalling and aggregation’ pathways. There were many significantly downregulated pathways, including RNA metabolism, DNA replication, and TP53 activity (FDR < 0.05; Fig. 1d and 1e, Supplementary Table S2 and S3).

We applied a stringent filtering approach based on both average gene expression and absolute logFC values considering up to 30 candidate genes (see Methods section) to identify more robust candidates of LC disease. This resulted in 27 unique candidate genes (Supplementary Table S4 and Supplementary Fig. S5), where most (18) were upregulated in LC cases compared to FalsePos, one of which was a gene with limited annotation (*ENSG00000259753*).

Cox models with LC death as endpoint were used to investigate associations of high versus low expression of the 26 annotated candidate genes to patient survival for different stage groups in the diagnostic study. There were eight candidate genes that were significantly associated with LC survival (FDR < 0.05, Benjamini–Hochberg method): i) *ANXA3, ARG1,* haptoglobin (*HP*)*,* integrin subunit alpha 2b (*ITGA2B*)*,* neuregulin 1 (*NRG1*) and oleoyl-ACP hydrolase (*OLAH*) with late-stage LC, with hazard ratios (HRs) 2.81, 2.16, 2.54, 2.16, 2.07 and 2.21 and 95% confidence intervals (CIs) 1.50–5.27, 1.20–3.90, 1.39–4.63, 1.21–3.86, 1.16–3.68 and 1.23–3.97, respectively, and ii) *ANXA3,* FAM20A golgi associated secretory pathway pseudokinase (*FAM20A*)*,* and long intergenic non-protein coding RNA 402 (*LINC00402*) with middle-stage LC, with HRs 2.48, 4.17 and 0.26 and 95% CIs 1.11–5.54, 1.72–10.12 and 0.10–0.64, respectively (Fig. 1g-i and Supplementary Fig. S6).

### Evaluation of candidate genes in the prospective studies

Out of the 26 annotated candidate genes, only 14 could be detected in the gene expression datasets of both prospective studies (NOWAC and HUNT3) generated in this work, mainly due to too low signal intensities in the microarray dataset (NOWAC; see Supplementary Methods for filtering criteria), and these were: adenylate kinase 5 (*AK5*)*, ARG1, ANXA3,* hexokinase domain containing 1 (*HKDC1*)*, HP,* indoleamine 2,3-dioxygenase 1 (*IDO1*)*, ITGA2B,* integrin subunit beta 3 (*ITGB3*)*,* myosin light chain 9 (*MYL9*)*, NRG1, OLAH,* phosphoglucomutase 5 (*PGM5*)*,* pro-platelet basic protein (*PPBP*), and transmembrane protein 176A (*TMEM176A*) (Supplementary Fig. S6). We correlated expression values (log_2_RPM) of these candidate genes in all the studies (Fig. 1f and Supplementary Fig. S7) and observed three strongly correlated clusters of genes: (i) *PPBP, MYL9, ITGA2B, ITGB3*, (ii) *ARG1, ANXA3, HP, OLAH* and (iii) *HKDC1, AK5*.

**Figure 2:**
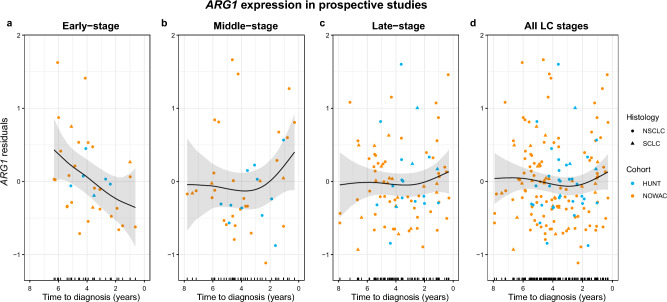
Time trends of *ARG1* expression in the prospective studies (NOWAC and HUNT3). (**a**) *ARG1* expression in early-stage, (**b**) middle-stage (**c**) late-stage and (**d**) all LC stages in relation to time between specimen collection and LC diagnosis. The time trends are visualized using smoothing splines from generalized additive models (gam, df = 2). As input to these models, we used residuals from mixed models (lmer) that were adjusted for sex, age (scaled) and smoking variable in addition to using study as random effect. Histological subtypes in this plot are visualized using different point shapes (NSCLC = non-small cell LC, SCLC = small cell LC) and the prospective studies are visualized with different point colours.

In the prospective studies, we first evaluated case–control differences for the candidate genes and observed significant association with LC only for one candidate (*TMEM176A*) for all LC and NSCLC cases compared to all controls (OR = 1.19 for both; 95% CI = 1.03–1.37; 1.03–1.38, respectively) (Supplementary Table S5). Except for *TMEM176A*, only three genes (*ANXA3*, *ARG1* and *HP*) showed consistently > 10% change in risk in all three comparisons (all LC cases vs. FalsePos, NSCLC cases vs. FalsePos and late-stage LC cases vs. FalsePos), though these were statistically significant only for all LC cases versus FalsePos without smoking adjustment (Supplementary Table S5). Low effect sizes in these comparisons of all cases and controls were not surprising, as blood specimens from cases in the prospective studies were collected from seemingly healthy individuals up to 8 years prior to LC diagnosis and some gene expression changes in prospective LC cases towards diagnosis would be expected. Therefore, we evaluated potential trends in candidate gene expression in relation to time between specimen collection and LC diagnosis for prospective LC cases only. These time to diagnosis models indicated a trend of slightly higher expression close to LC diagnosis for the candidate gene *ARG1*, noticeable up to about two years prior to the LC diagnosis for middle- and late-stage LC cases (Fig. 2b and 2c). Of note, a trend of lower *ARG1* expression with closer time to LC diagnosis was observed for early-stage LC cases (Fig. 2a). *ANXA3* showed a similar trend of higher expression in the last two years prior to LC diagnosis, as for *ARG1* (Supplementary Fig. S7). For the other candidate genes, no significant trend in relation to the time of diagnosis could be observed for prospective LC cases. Based on the higher expression of *ANXA3* and *ARG1* close to LC diagnosis indicated in the time to diagnosis models, we restricted the case–control analysis of these two candidates to the last two years prior to LC diagnosis. We found significant associations for *ANXA3* and *ARG1* with LC in all the three comparisons (all LC, NSCLC and late-stage LC cases compared to all controls) (Supplementary Table S6). The strongest associations were observed for *ARG1* in late-stage LC cases (OR = 5.01, 95% CI 1.89–13.30, * p*-value = 1.22E-03).

### Blood cell type estimates

As one of the most highly upregulated pathways in the Reactome analysis was neutrophil degranulation for all the three comparisons with DE genes in the diagnostic study, especially in late-stage LC cases (Fig. 1d), we wanted to elucidate if changes in blood cell type proportions would influence the associations of candidate genes with LC. As blood counts were not available in this study, we estimated cell type proportions derived from both blood RNA-Seq and DNAm data generated for the same individuals included in this work (unpublished DNAm data; see Methods section and Supplementary Methods for details). Further, we evaluated if the blood cell type estimates are consistently reported as significantly different between cases and FalsePos for the proportions estimated from DNAm and RNA-Seq data. Only blood estimates of CD4 T cells, NK cells and neutrophils were significantly associated with LC at diagnosis (Supplementary Table S7 and S8).

In the prospective studies, blood cell type estimates were obtained similarly from both RNA-Seq/microarray data and DNAm data as for the individuals from the diagnostic study (see Methods section and Supplementary Methods for details). However, no significant association of CD4 T cells, NK cells and neutrophils with LC could be observed in the prospective studies (Supplementary Table S7 and S8).

### LC discrimination based on candidate genes and blood cell type estimates

**Figure 3:**
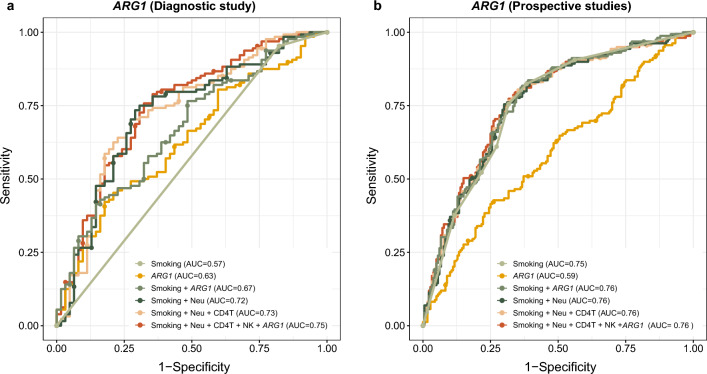
Receiver operating characteristic (ROC) curves in the diagnostic and prospective studies for the *ARG1* candidate. (**a**) LC discrimination based on 128 cases and 62 FalsePos in the diagnostic study (NLCB). (**b**) LC discrimination based on 163 cases and 184 controls in the prospective studies (NOWAC and HUNT3). Six separate ROC curves are visualized for models including (i) smoking status/ variable only (“Smoking”), (ii) *ARG1* expression only (“*ARG1*”), (iii) both the smoking status/ variable and *ARG1* expression (“Smoking + *ARG1*”), (iv) both the smoking status/ variable and blood neutrophil estimates (“Smoking + Neu”), (v) the smoking status/ variable, blood neutrophil and CD4 T cell estimates (“Smoking + Neu + CD4T”) and (vi) the smoking status/ variable, blood neutrophil, CD4 T cell and NK cell estimates and candidate gene expression (“Smoking + Neu + CD4T + NK + *ARG1*”). ROC curves of the smoking status/ variable, blood neutrophil, CD4 T cell and NK cell estimates (“Smoking + Neu + CD4T + NK”) are not shown here, as they were very similar to the “Smoking + Neu + CD4T” ROC curves with almost identical AUC values. In the diagnostic study, smoking status was defined as never/ever smokers and in the prospective studies as smoking variable with seven categories combining smoking status (never/current/former) and pack-years (see Methods section for details).

We also tested the ability of the 14 candidate genes to discriminate between LC cases and controls/FalsePos. In the diagnostic study, the candidate genes alone were able to discriminate LC cases from FalsePos better compared to smoking status (ever/never) alone (AUC = 0.63 and 0.57, respectively, for *ARG1* as an example in Fig. 3). When including both the candidate gene and smoking status in the model, the discriminative ability slightly improved (AUC = 0.67 for *ARG1*; Fig. 3). When including blood neutrophil estimates together with smoking status, the discriminative ability was higher (AUC = 0.72), and marginally improved by adding CD4 T cell and NK cell estimates to the model (AUC = 0.73 for both). The LC discriminative ability further slightly improved by including the candidate gene *ARG1* in the model (AUC = 0.75; Fig. 3). However, the most significant effects in LC discrimination were observed by including blood neutrophil estimates in the models (Fig. 3 and Supplementary Fig. S9). Similar observations were made for the other candidates (Supplementary Fig. S9).

In the prospective studies, the discriminative ability of the candidate genes alone was lower than of the smoking variable (seven categories) alone (AUC = 0.59 and 0.75, respectively, for *ARG1* as an example in Fig. 3). The LC discrimination improved only marginally when both the candidate gene and the smoking variable were included in the model (AUC = 0.76 for *ARG1*; Fig. 3) and remained the same when additionally including neutrophil, CD4 T cell and NK cell blood estimates or the candidate gene *ARG1* in the model (AUC = 0.76; Fig. 3). The ROC curves and AUC values were similar to *ARG1* (*TMEM176A* and *NRG1*, AUC = 0.59 for both; *ANXA3*, AUC = 0.58) or lower for the other candidate genes in the prospective studies (Supplementary Fig. S9). Overall, both the blood cell type estimates and the candidate genes improved the LC discrimination in the diagnostic study compared to the smoking status information (never/ever) alone (Fig. 3 and Supplementary Fig. S9). However, in the prospective studies, discriminative ability of candidate genes did not improve beyond that of the smoking variable information, nor by adding the blood cell type estimates (Supplementary Fig. S9).

## Discussion

We aimed to identify candidate genes associated with LC in whole blood specimens taken at LC diagnosis using genome-wide expression profiling and evaluate their associations with LC in two prospective studies. Case–control comparisons and further filtering identified 14 candidate genes in the diagnostic study. High expression of three candidate genes (*ANXA3*, *ARG1* and *HP*) was found to be strongly associated with survival of late-stage LC cases in the diagnostic study (FDR values 0.009, 0.03, and 0.007, respectively). In the prospective studies, only one of the candidates (*TMEM176A*) showed significant, though weak (OR = 1.19) association with LC in the case–control comparisons for all prospective LC or NSCLC cases with whole blood specimens up to eight years prior to LC diagnosis. However, high expression of two candidates, *ANXA3* and *ARG1*, was strongly associated with LC, and especially with late-stage LC in the last two years prior to LC diagnosis (ORs 3.47 and 5.00, respectively).

In the diagnostic study, we observed significant gene expression differences only for all LC, late-stage LC and NSCLC cases compared to controls (FalsePos). The predominantly enriched pathway was neutrophil degranulation, which was upregulated in LC cases. As differential gene expression studies in whole blood are typically confounded by cell type composition changes^23^, we further investigated if blood cell type estimates based either on blood DNA methylation or gene expression data were associated with LC. As expected, the blood neutrophil estimates, together with CD4 T cell and NK cell estimates, were significantly associated with LC at diagnosis, supporting earlier studies that an elevated neutrophil-to-lymphocyte ratio has a prognostic value at^24,25^ and potentially few months prior to LC diagnosis^26^ – an observation primarily driven by neutrophils^50^. Including blood cell type estimates, and especially blood neutrophils, together with the candidate genes in the ROC curve analysis strongly improved the LC discriminative ability at diagnosis compared to smoking status alone.

Higher expression of *ANXA3* and *ARG1* has been significantly associated with lower survival among late-stage LC cases^51,52^, but association with LC the last two years prior to LC diagnosis is novel. However, these two genes did not considerably improve the LC discriminative ability for prospective LC cases beyond the smoking information available in this work.

Overexpression of *ANXA3* has been previously linked to tumour proliferation and metastasis in many tumours, including LC^53^. *ANXA3* was reported to have both diagnostic and prognostic potential in several cancers, including LC^54^, but only studies with small number of participants have been performed so far^53^. In this work, high *ANXA3* expression was associated with lower survival in late-stage LC cases, which is in agreement with previous work^55^. However, the association of *ANXA3* expression with LC remains controversial, as both up- and downregulation in LC tissue have been observed^53^. Although high blood *ANXA3* expression has been associated with different cancer types^52,53^ and interestingly, *ANXA3* was included in a blood-based 7-gene biomarker panel for colorectal cancer discrimination^56^, this work appears to be the first one reporting high *ANXA3* expression in whole blood of LC patients.

High levels of *ARG1* are associated with cell cycle arrest and functional unresponsiveness in T cells^57^. High *ARG1* expression has been observed in infections, including severe covid-19 patients^58^, and many human cancers, both in tumour tissue and in peripheral blood^51^, including LC^59^. High *ARG1* expression seems to be a negative predictive factor in many cancers and correlates with a more aggressive phenotype^51^. In line with these reports, we observed a trend of higher *ARG1* expression in the last two years prior to LC diagnosis in late-stage cancer cases. However, high amount of intratumoural *ARG1*+ neutrophils might not always be associated with worse cancer prognosis^60,61^. It appears that combining *ARG1*-targeting vaccines with anti-PD-1 immunotherapy increases T cell infiltration in tumours^62^ (NCT03689192). Interestingly, there is a large ongoing clinical trial for arginase inhibitor INCB001158 as a single agent or in combination with immunotherapy in patients with advanced or metastatic solid tumours (NCT02903914). Of note, we observed a trend of lower *ARG1* expression towards LC diagnosis for early-stage cases. Although this is an interesting observation, it needs to be followed-up in other work, since this dataset included only few early-stage cases. As upregulation of both *ARG1* and *ANXA3* has been previously observed in several other diseases^51,52^, they are likely not specific to LC, but their higher expression could rather be associated with disease severity. Still, it is not known whether these associations represent signals linked to the disease development or systemic responses to the disease.

*TMEM176A* and *HP* were two other relevant candidate genes associated with LC in this work. *TMEM176A* expression was overall significantly (FDR < 0.05) associated with prospective LC cases, though weakly (OR = 1.19). Although upregulation of *TMEM176A* has previously been observed in NSCLC tissue^63^, it might act as a tumour suppressor gene in colorectal and esophageal cancer tissues^64,65^. We are not aware of any reported associations with LC in peripheral blood. *HP* expression was significantly associated with survival of late-stage LC cases in the diagnostic study in this work, which is in agreement with previous studies based on both peripheral blood^66,67^ and tissue specimens^68^. Higher serum haptoglobin protein (Hp) levels were also previously associated with advanced LC and poor prognosis in NSCLC patients^69^. Associations of the candidate genes with LC did not change considerably before and after adjustment for smoking, suggesting that these markers are unrelated to tobacco exposure. In addition, none of the 14 candidate genes was previously found associated with smoking^70^.

Interestingly, compared to other similar gene expression studies^15,27^, the candidate markers of LC found in our study (*ANXA3, ARG1* and *HP*) were observed significantly upregulated (FDR < 1.55E-04) in a lung cancer subtype defined as ‘LC2’ compared to healthy controls in the study performed by Zhang and colleagues^15^ (with logFC values 0.69, 0.79 and 1.38, respectively). In addition, four of our candidate genes (*AK5*, *IDO1*, *NRG1* and *OLAH*) were also found DE in that study, where all except *IDO1* had the same effect direction. Further, we observed two of our candidate markers (*ANXA3* and *ARG1*) significantly upregulated (FDR < 4.82E-02) in NSCLC cases compared to the controls in the training set (TS) dataset in the study performed by Zander and colleagues^27^ (with logFC values 0.70 and 1.09, respectively) when adjusted for age (scaled) and sex (all individuals were ever smokers). Additionally, all except one (*HKDC1*) of our 14 candidate genes were detected with the same effect direction in NSCLC cases compared to the controls in that study (p = 0.002; binomial test).

As several LC screening trials have been conducted or are in progress, the length of the screening interval is being discussed and tested^71,72^. Biennial screening rounds for high-risk individuals have been suggested as the most promising strategy avoiding high percentage of interval cancers and reducing unnecessary radiation exposure^71,72^. These screenings can potentially be complemented with a blood biomarker panel to improve the overall LC screening performance in a personalized strategy, as documented by Pastorino and colleagues^73^. The present results further support that systemic gene expression changes indicating cancer progression may be detected in whole blood already prior to LC diagnosis.

The main strength of this work lies in combining a diagnostic study for exploratory analysis leading to identification of LC candidates that were then evaluated in two prospective studies. This is a unique approach to identification of potential cancer candidate markers. Through this approach we could also evaluate expression trends of the candidate genes in individuals prior to LC diagnosis. In addition, the diagnostic study included symptomatic individuals (FalsePos) instead of healthy controls, which resembles a screening situation. Further, we included all subtypes and LC stages in this work.

This work has some limitations that should be addressed in future studies. Although we initially identified 27 unique candidate genes in the diagnostic study based on RNA-seq analysis, one gene was excluded due to limited annotations and 12 genes were excluded because of too low signal intensities in the microarray dataset (NOWAC study), as the majority (72%) of the prospective specimens in this work were from the NOWAC study. However, these 12 genes could potentially be relevant genes associated with LC, and future studies should ideally use RNA-seq instead of microarrays for genome-wide expression analyses. In addition, expression differences in the prospective studies could have been affected by different sampling systems, as blood specimens in the NOWAC and HUNT3 studies were collected using PAXgene and Tempus tubes, respectively^74^. Further, smoking information in the diagnostic study was not found to be reliable beyond smoking status, in contrast to the more detailed smoking information available in the prospective studies. Therefore, not adjusting for a more detailed information of smoking habits could have resulted in a residual confounding by smoking in the diagnostic study analysis, and potentially also explain why we observe better LC discriminative ability of the candidate gene markers in the ROC curve analysis in the diagnostic study compared to prospective studies. In addition, the time to diagnosis analyses in the prospective studies have been performed on different individuals with different time to LC diagnosis. No longitudinal analyses of same individuals could be performed since whole blood specimens of the included individuals were not available at multiple time points prior to LC diagnosis.

In summary, we identified 14 LC candidate genes from whole blood specimens using both diagnostic and prospective studies. High expression of several candidate genes was strongly associated with lower LC survival in the diagnostic study, and the strongest associations were observed for *ANXA3* and *ARG1* in prospective late-stage LC cases, especially within two years of LC diagnosis. Blood cell type estimates of CD4 T cells, NK cells and especially neutrophils were strongly associated with LC in the diagnostic study and improved the LC discriminative ability at diagnosis beyond inclusion of the candidate genes.

## Conclusion

Our results indicate that increased expression of *ANXA3, ARG1* and *HP* can be detected in whole blood specimens both at and close to LC diagnosis beyond LC-associated changes in blood cell type proportions, and that these genes represent diagnostic and prognostic markers of late-stage LC.

### Supplementary Information

Supplementary Information 1.

Supplementary Table S2.

Supplementary Table S3.

## Data Availability

The normalized count matrix of expressed genes in the NLCB study generated during the current study is available in the Gene Expression Omnibus (GEO) repository, [GSE198048, https://www.ncbi.nlm.nih.gov/geo/query/acc.cgi?acc=GSE198048]. The raw microarray and sequencing data generated in the prospective studies can be accessed upon reasonable request to the originating cohorts (for data from HUNT cohort to kontakt@hunt.ntnu.no and for data from NOWAC cohort to NOWAC@uit.no). Access will be conditional to adherence to local ethical and security policies. R codes used for the data analyses in this article are available from the corresponding author upon reasonable request.

## Abbreviations

AD: Adenocarcinoma
AUC: Area under the curve
DNAm: DNA methylation
edNeu: Gene expression-derived neutrophil proportions
edNLR: Gene expression-derived neutrophil-to-lymphocyte ratio
FalsePos: Individuals with suspected LC but negative LC diagnostic evaluation
FC: Fold change
FDR: False discovery rate
HUNT3: The Trøndelag health study 3
LC: Lung cancer
mdNeu: Methylation-derived neutrophil proportions
mdNLR: Methylation-derived neutrophil-to-lymphocyte ratio
NLCB: The Norwegian lung cancer biobank
NLR: Neutrophil-to-lymphocyte ratio
NOWAC: The Norwegian women and cancer study
NSCLC: Non-small cell lung cancer
OR: Odds ratio
RPM: Reads per million
ROC curve: Receiver operating characteristic curve
RPC: Robust partial correlations
RNA-seq: RNA sequencing
SCLC: Small cell lung cancer
SQ: Squamous cell carcinoma
