# Supplementary material for: Increased expression of individual genes in whole blood is associated with late-stage lung cancer at and close to diagnosis
Source: Sci Rep. 2023 Nov 25;13:20760. doi: 10.1038/s41598-023-48216-z (PMC10676373; doi:10.1038/s41598-023-48216-z)
Supplement: Supplementary file 1 — Supplementary Information 1. [file 41598_2023_48216_MOESM1_ESM.pdf]

Supplementary Information

Increased expression of individual genes in whole blood is associated with late-stage lung cancer at and close to diagnosis

Ilona Urbarova[1] *, Anne Heidi Skogholt[2], Yi-Qian Sun[3,4,5], Xiao-Mei Mai[2], Bjørn Henning Grønberg[3,6], Torkjel Manning Sandanger[1], Pål Sætrom[2,3,6,7]**, Therese Haugdahl Nøst[1,2]**

[1]Department of Community Medicine, Faculty of Health Sciences, UiT The Arctic University of Norway, Tromsø, Norway;

[2]Department of Public Health and Nursing, Norwegian University of Science and Technology, Trondheim, Norway;

[3]Department of Clinical and Molecular Medicine, NTNU, Norwegian University of Science and Technology, Trondheim, Norway;

[4]Department of Pathology, Clinic of Laboratory Medicine, St. Olavs Hospital, Trondheim University Hospital, Trondheim, Norway;

[5]Center for Oral Health Services and Research Mid-Norway (TkMidt), Trondheim, Norway;

[6]Department of Oncology, St. Olavs Hospital, Trondheim University Hospital, Trondheim, Norway;

[7]Bioinformatics Core Facility, Norwegian University of Science and Technology, Trondheim, Norway;

* Corresponding author: Ilona Urbarova, UiT The Arctic University of Norway, P.O. Box 6050 Langnes, NO-9037 Tromsø, Norway; E-mail: ilona.urbarova@uit.no

** These authors jointly supervised this work

Supplementary Methods

Norwegian Lung Cancer Biobank (NLCB)

The Norwegian Lung Cancer Biobank (NLCB) is a hospital-based biobank that was established in 2005 at St. Olavs Hospital, Trondheim, Norway. This biobank is managed by Biobank1, a collaboration between NTNU and hospitals in the region of middle Norway that manage regional clinical biobanks. Inclusion criteria to the NLCB biobank are: (i) patients with suspected LC with a biopsy or blood specimens taken, (ii) patients with LC relapse, (iii) patients aged 18 years or older, (iv) written informed consent, (v) sampling does not imply significant delay of treatment, and (vi) sampling does not imply a significantly increased risk compared to routine diagnostic sampling. The recruited participants included individuals consecutively admitted to hospital and suspected to have LC. Thus, patients with and without LC were included. Symptomatic persons with a positive CT scan that were suspected of LC, but were confirmed negative by histological verification are referred to as 'false positives' (FalsePos). LC cases had histologically verified LC of any histological subtype and stage, as they were collected during clinical evaluation for lung cancer, and therefore comprises of a random sample of early-, middle- and late-stage cases. The blood specimens were taken during diagnostic evaluation before the individuals received any treatment. Specimens from lung and blood were collected together with phenotype data. Whole blood specimens were collected using RNA stabilizing tubes (PAXgene) at recruitment into the study during diagnostic evaluation and prior to any treatment, and stored at -80°C. Phenotype data was collected from questionnaires and electronic health records from the hospital. None of the patients had started cancer treatment at the time of sampling. In total, 62 individuals were classified as FalsePos.

Prospective studies

This work includes pre-diagnostic specimens from two prospective studies from adult populations in Norway: the Norwegian Women and Cancer Study (NOWAC; the whole Norway) and the Trøndelag Health Study 3 (HUNT3; Trøndelag county in the middle of Norway).

NOWAC is a nationally representative cohort initiated in 1991 at UiT The Arctic University of Norway[1]. Women aged 30-70 years were randomly selected from the National Registry and invited to participate in the study. Participants filled out a questionnaire at recruitment and have been followed-up with up to three questionnaires since then. The questionnaires have covered self-reported anthropometry and lifestyle variables, including detailed information on past and current smoking habits. A case-control study nested within the NOWAC cohort was conducted among participants who had donated a blood specimen in 2003-2006 (N = 48,941). A one-page

questionnaire including information about recent and current smoking habits was also filled out at the time of blood donation. Blood specimens were collected in PAXgene tubes and stored at −80°C. Through linkage with the Cancer Registry of Norway we identified 134 participants who had been diagnosed with LC after they donated a blood specimen. For each case, one cancer-free control was randomly drawn from NOWAC participants with available blood specimens and matched on birth year and blood specimen collection batch.

The HUNT Study is a population-based health survey established at NTNU[2]. All inhabitants aged 20 years or older in the northern area of Trøndelag have been invited to four surveys: HUNT1 (1984–1986), HUNT2 (1995–1997), HUNT3 (2006–2008) and HUNT4 (2017–2019). More than 120,000 individuals have participated and responded to questionnaires and donated blood specimens. A nested case-control study was designed within participants in the HUNT3 survey, including 38 incident cases who developed LC after donating blood specimens in 2006-2008 and 58 controls matched on sex and age (±3 years). Incident LC cases were identified using a linkage of data between HUNT and the Cancer Registry of Norway. Blood specimens were collected in Tempus blood RNA tubes and stored at -80°C after blood collection. Information on lifestyle variables, including smoking habits, was extracted from the questionnaires.

RNA isolation, sequencing and data processing in NLCB study

Total RNA was extracted from PAX tubes using the Qiagen PAXgene Blood miRNA Kit (Qiagen, MD, USA) in combination with an automated Qiacube system according to manufacturer's instructions. RNA quantity, quality and purity were evaluated using Qubit (Thermo Fisher Scientific, MA, USA), Bioanalyzer (Agilent, CA, USA) and NanoDrop (Thermo Fisher Scientific, MA, USA). 400 ng of total RNA was then incubated with magnetic beads coated with oligo-dT, then all other RNAs except polyadenylated RNAs were removed by washing. Isolation and purification of the polyadenylated RNA was performed at Biobank1, St. Olavs Hospital, Trondheim, Norway. RNA sequencing libraries for specimens from NLCB study were generated using the Lexogen SENSE mRNA-Seq library prep kit according to manufacturer's instructions (Lexogen GmbH, Vienna, Austria). In brief, library preparation was initiated by random hybridization of starter/stopper heterodimers to the poly(A) RNA still bound to the magnetic beads. These starter/stopper heterodimers contain Illumina-compatible linker sequences. A single-tube reverse transcription and ligation reaction extends the starter to the next hybridized heterodimer, where the newly-synthesized cDNA insert was ligated to the stopper. Second-strand synthesis was performed to release the library from the beads. The resulting double-stranded library was purified and amplified (12 PCR cycles) after adding the adaptors and indexes. Finally, libraries were quantitated by qPCR using KAPA Library Quantification Kit (Kapa Biosystems, Inc., MA, USA) and validated using Agilent High Sensitivity DNA Kit on a Bioanalyzer

(Agilent Technologies, CA, USA). The size range of the DNA fragments were measured to be in the range of 200-600 bp with average library size 300 bp.

Prior to sequencing, the libraries were quantified (KAPA Library Quantification Kit (Illumina/ABI Prism), normalized and pooled. Quantitated libraries were further diluted to 2.3 pM and subject to clustering by a cBot Cluster Generation System prior to sequencing on one HiSeq4000 flowcell (Illumina Inc. San Diego, CA, USA), according to manufacturer's instructions. Finally, single-end read sequencing was performed for 50 cycles on an Illumina HiSeq4000 instrument, in accordance with the manufacturer's instructions (Illumina, Inc., San Diego, CA, USA).

FASTQ files were created using bcl2fastq 2.17 (Illumina, Inc., San Diego, CA, USA). The sequencing reads in the individual FASTQ files from NLCB study were trimmed and quality filtered using cutadapt v1.18[3] with additional options *-j 10 -a AGATCGGAAGAGCACACGTCTGAACTCCAGTCAC -q 15,10 -m 14*, mapped to the human genome (hg38) using STAR aligner v2.7.7a[4] with additional options *--chimSegmentMin 30 --outFilterMultimapNmax 20 --alignSJoverhangMin 8 --alignSJDBoverhangMin 1 --outFilterMismatchNmax 10 --outFilterMismatchNoverLmax 0.04 --alignIntronMin 20 --alignIntronMax 100000* and counted using *featureCounts* v2.0.1[5] with additional options *-O --largestOverlap --primary --ignoreDup -s 2 -J*. Resulting count matrix including all specimens was used for the statistical analyses. The count matrix was annotated using *AnnotationDbi* R package v1.52.0[6] (*org.Hs.eg.db* human database) and filtered based on RPM values using following filtering criteria: *rowSums(RPM(matrix)) > ncol(matrix) * 2*, resulting in a matrix with 14,014 Ensembl annotated genes. Exploratory analyses were done using Principal Component Analysis (PCA) plot and revealed DNA contamination and read ambiguity of the specimens (Supplementary Fig. 1a and 1b). DNA contamination was estimated using total amount of reads assigned in stranded vs. unstranded read count summary, and read ambiguity was estimated using total counts per specimen vs. total amount of reads assigned in stranded read count summary. These estimated ratios were included as a combined 'technical variation' covariate in limma models used for the statistical analyses. Principal component analysis (PCA) plots did not show any clear separation of cases from FalsePos based either on the LC stage or the histological subtype (Supplementary Fig. 1c and 1d).

RNA isolation and sequencing in the HUNT3 study

Total RNA in Tempus RNA blood tube was extracted using Maxwell® 16 LEV simplyRNA Blood Kit (Promega) according to the manufacturer's instructions. The blood-derived RNA specimens were treated with DNase and the integrity was assessed using Agilent RNA 6000 Pico Kit on a 2100 Bioanalyzer instrument (Agilent Technologies,

Santa Clara, CA, USA). RNA sequencing libraries were prepared using Illumina TruSeq® Stranded Total RNA with Ribo-Zero™ Globin kit (Illumina, San Diego, CA, USA) according to the manufacturer's instructions. In brief, 1200 ng total RNA was used as starting material. The first step involved the removal of globin-encoding mRNA in addition to the ribosomal RNA, using biotinylated target-specific oligos combined with Ribo/Globin-Zero RNA removal beads. Following purification, the RNA was fragmented using divalent cations at 94°C for 3 min. First and second strand cDNAs were synthesized using random oligonucleotides and SuperScript II, followed by DNA polymerase I and RNase H. Exonuclease and polymerase was used to produce blunted overhangs. Illumina SR adapter and index oligonucleotides were ligated to the cDNA after 3' end adenylation. DNA fragments were enriched by 15 cycles of PCR reaction. The libraries were purified using the AMPure XP (Beckman Coulter, Inc., Indianapolis, IN, USA), quantitated by qPCR using KAPA Library Quantification Kit (Kapa Biosystems, Inc., Wilmington, MA, USA) and validated using Agilent High Sensitivity DNA Kit on a Bioanalyzer (Agilent Technologies, Santa Clara, CA, USA). The sizes of the DNA fragments were measured to be in the range of about 210–400 bp and peaked around 290 bp. Quantitated libraries were further diluted to 2.8 nM and subjected to clustering by a cBot Cluster Generation System on two HiSeq4000 flowcells (Illumina Inc. San Diego, CA, USA), according to manufacturer's instructions. Finally, single-end read sequencing was performed for 75 cycles on an Illumina HiSeq4000 instrument, in accordance with the manufacturer's instructions (Illumina, Inc., San Diego, CA, USA). FASTQ files were created with bcl2fastq v2.17 (Illumina, Inc., San Diego, CA, USA). Transcript expression values were generated by quasi alignment using salmon[7] and the Ensembl human reference genome (Ensembl, GRCh38, release 92). Aggregation of transcript to gene expression was performed using R Bioconductor package *tximport* and normalized to reads per million (RPM) values.

RNA isolation, microarray analyses and data processing in NOWAC study

RNA isolation, quality control, hybridization experiments and initial post-processing analyses were performed at the Genomics Core Facility (GCF), Norwegian University of Science and Technology (NTNU), Trondheim, Norway. Briefly, total specimen RNA was isolated from the whole blood specimens in PAXgene tubes using established protocols[8]. Specimens were analyzed similarly as previously reported [9,10] using the IlluminaHuman HT-12 expression bead chips and Illumina GenomeStudio 1.9.0 was used to assess the quality of each array. Of the 268 specimens analyzed, 17 case–control pairs were excluded due to laboratory quality measures before original probe values were background corrected using negative controls (function *nec*, R package *limma*)[11]. Further, probes reported to have poor quality from Illumina, no annotation or detected in <10% of specimens were removed and values were quantile normalized (R *lumi:lumiN*) and $\log_2$ transformed (R *lumi:lumiT*)[12]. Annotation

of pre-processed data was obtained using R *lumi:nuID2RefSeqID* and *illuminaHumanv4.db*[13] packages. The statistical analyses were performed using 11,483 annotated and unique genes in 125 cases and 126 controls.

DNA methylation data and processing

NLCB study

Genome-wide DNA methylation profiles from bisulphite-converted, hybridized genomic DNA from buffy coat specimens were generated using Illumina Infinium MethylationEPIC BeadChips, similarly as described elsewhere[14]. Briefly, probes (i) reported to be cross-hybridizing probes[15], (ii) out-of-band, and (iii) with at least one CpG with detection p-value above 0.8 were removed. The final matrix used in this work included 866,091 DNA methylation sites in 178 specimens (126 cases and 52 controls).

NOWAC study

Genome-wide DNA methylation profiles from bisulphite-converted, hybridized genomic DNA from buffy coat specimens were generated using Illumina Infinium HumanMethylation450 Bead-Chips as previously described[16]. DNA methylation levels at each locus were expressed as the ratio of intensities arising from methylated cytosines over total intensities. Specimen preparation and data pre-processing were performed as described elsewhere[16]. In brief, probes (i) on sex chromosomes, (ii) reported to be cross-reactive[17] and (iii) for which methylation levels were measured in <20% of the specimens were excluded. The final matrix used in this work included 428,629 probes targeting autosomal CpG loci in 251 women (125 cases and 126 controls).

HUNT3 study

Genome-wide DNA methylation profiles from bisulphite-converted, hybridized genomic DNA from buffy coat specimens over 850 K DNA methylation sites were quantified using the Illumina Infinium MethylationEPIC BeadChip kit as previously described[18]. Specimen preparation and data pre-processing were performed as described elsewhere[18]. Briefly, probes (i) on sex chromosomes and (ii) considered as outliers from the methylated and unmethylated signal comparison were excluded. Specimens were normalized based on internal control probes and principal components to remove technical batch effects. The final matrix used in this work included 864,674 DNA methylation sites in 92 specimens (55 controls and 37 cases).

Statistics and reproducibility

All statistical analyses in this article were performed using R programming language v4.0.5. The number of independent biological samples and type of statistical tests used are described in detail in the respective sections of Methods or in Supplementary Methods.

Supplementary References

1. Lund, E. *et al.* Cohort profile: The Norwegian Women and Cancer Study--NOWAC--Kvinner og kreft. *Int. J. Epidemiol.* **37**, 36-41, doi:10.1093/ije/dym137 (2008).

2. Krokstad, S. *et al.* Cohort Profile: the HUNT Study, Norway. *Int. J. Epidemiol.* **42**, 968-977, doi:10.1093/ije/dys095 (2013).

3. Martin, M. Cutadapt removes adapter sequences from high-throughput sequencing reads. *EMBnet. journal* **17**, 10-12 (2011).

4. Dobin, A. *et al.* STAR: ultrafast universal RNA-seq aligner. *Bioinformatics* **29**, 15-21, doi:10.1093/bioinformatics/bts635 (2013).

5. Liao, Y., Smyth, G. K. & Shi, W. featureCounts: an efficient general purpose program for assigning sequence reads to genomic features. *Bioinformatics* **30**, 923-930, doi:10.1093/bioinformatics/btt656 (2014).

6. Pagès, H., Carlson, M., Falcon, S. & Li, N. AnnotationDbi: Manipulation of SQLite-based annotations in Bioconductor. *R package version* **1** (2019).

7. Patro, R., Duggal, G., Love, M. I., Irizarry, R. A. & Kingsford, C. Salmon provides fast and bias-aware quantification of transcript expression. *Nat. Methods* **14**, 417-419, doi:10.1038/nmeth.4197 (2017).

8. Dumeaux, V. *et al.* Peripheral blood cells inform on the presence of breast cancer: a population-based case-control study. *Int. J. Cancer* **136**, 656-667, doi:10.1002/ijc.29030 (2015).

9. Nøst, T. H. *et al.* Transcriptomic signals in blood prior to lung cancer focusing on time to diagnosis and metastasis. *Scientific reports* **11**, 1-11 (2021).

10. Sandanger, T. M. *et al.* DNA methylation and associated gene expression in blood prior to lung cancer diagnosis in the Norwegian Women and Cancer cohort. *Scientific Reports* **8**, doi:10.1038/s41598-018-34334-6 (2018).

11. Shi, W., Oshlack, A. & Smyth, G. K. Optimizing the noise versus bias trade-off for Illumina whole genome expression BeadChips. *Nucleic Acids Res.* **38**, e204, doi:10.1093/nar/gkq871 (2010).

12. Günther, C.-C., Holden, M. & Holden, L. Preprocessing of gene-expression data related to breast cancer diagnosis. *Report SAMBA/35/14 Norwegian Computing Central available from: http://publications. nr. no/directdownload/directdownload/1415353311/preprocessinggunther.pdf. Accessed April* **17**, 2016 (2014).

13. Dunning, M., Lynch, A. & Eldridge, M. illuminaHumanv4. db: Illumina HumanHT12v4 annotation data (chip illuminaHumanv4). *R package version* **1** (2015).

14. Page, C. M. *et al.* Lifetime Ultraviolet Radiation Exposure and DNA Methylation in Blood Leukocytes: The Norwegian Women and Cancer Study. *Sci Rep* **10**, 4521, doi:10.1038/s41598-020-61430-3 (2020).

15. McCartney, D. L. *et al.* Identification of polymorphic and off-target probe binding sites on the Illumina Infinium MethylationEPIC BeadChip. *Genom Data* **9**, 22-24, doi:10.1016/j.gdata.2016.05.012 (2016).

16. Guida, F. *et al.* Dynamics of smoking-induced genome-wide methylation changes with time since smoking cessation. *Hum. Mol. Genet.* **24**, 2349-2359, doi:10.1093/hmg/ddu751 (2015).

17. Price, M. E. *et al.* Additional annotation enhances potential for biologically-relevant analysis of the Illumina Infinium HumanMethylation450 BeadChip array. *Epigenetics Chromatin* **6**, 4, doi:10.1186/1756-8935-6-4 (2013).

18. Sun, Y. Q. *et al.* Assessing the role of genome-wide DNA methylation between smoking and risk of lung cancer using repeated measurements: the HUNT study. *Int. J. Epidemiol.* **50**, 1482-1497, doi:10.1093/ije/dyab044 (2021).

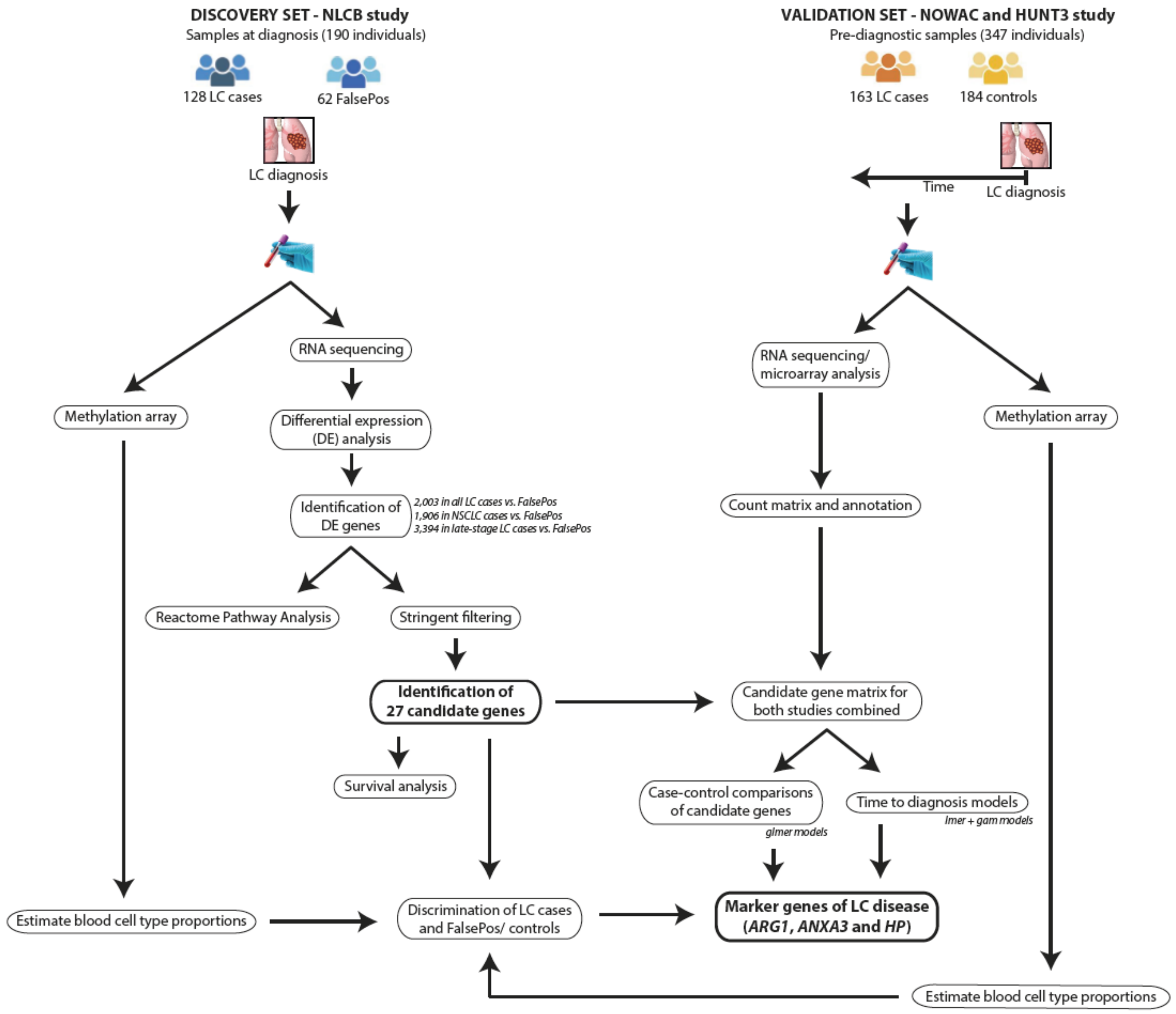

Supplementary Fig. S1. Analysis workflow. Visualized is the workflow diagram of our analysis. We performed differential expression (DE) analysis using the diagnostic study, i.e. NLCB study (discovery set), where we identified 27 candidate genes of lung cancer (LC). We evaluated the performance of these 27 candidate genes using the prospective studies, i.e. NOWAC and HUNT3 study (validation set) and validated three genes (*ARG1*, *ANXA3* and *HP*) as marker genes of LC disease.

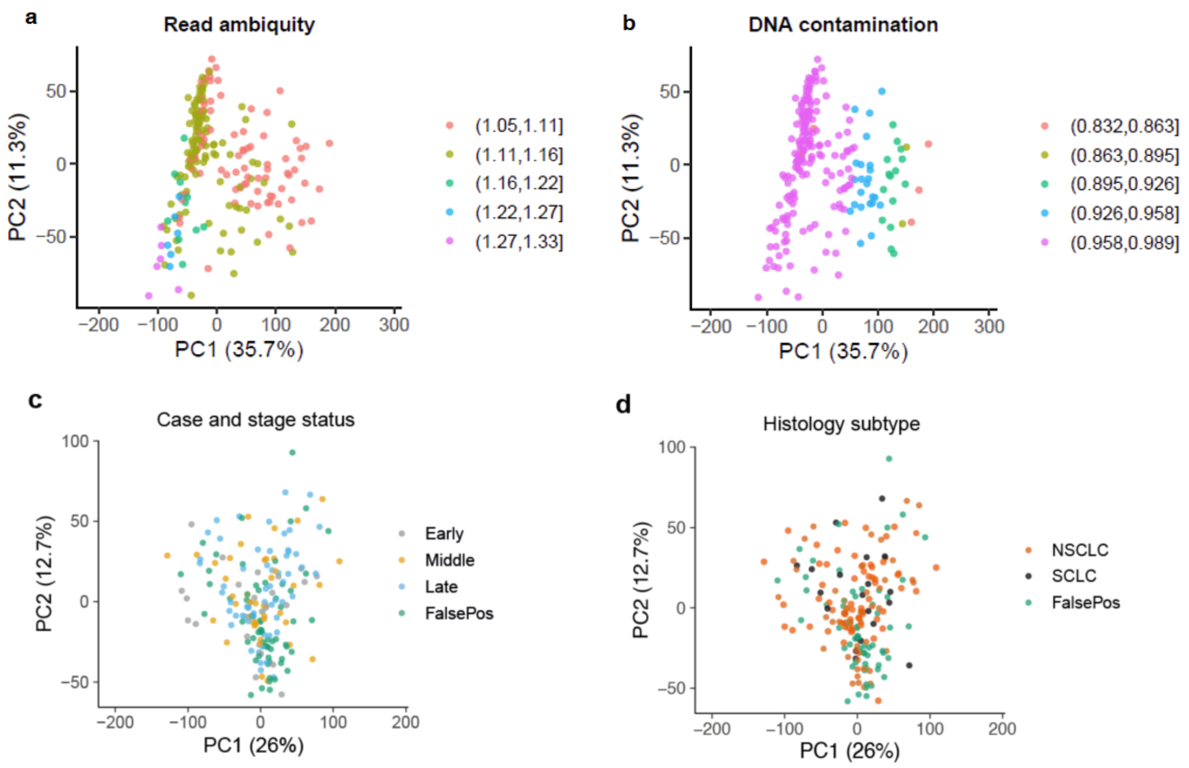

Supplementary Fig. S2. Clustering of the specimens in the diagnostic study. (a, b) Principal component analysis (PCA) plots showing read ambiguity (a) and DNA contamination (b) in gene expression data from whole blood specimens from individuals included in the diagnostic study (NLCB); these batch effects were included as a combined covariate in the linear models (limma) used for statistical analyses. (c, d) PCA plots after correction for technical variation as shown in (a, b) and coloured according to (c) case and stage status or (d) histology subtype. NSCLC = non-small cell LC, SCLC=small cell LC.

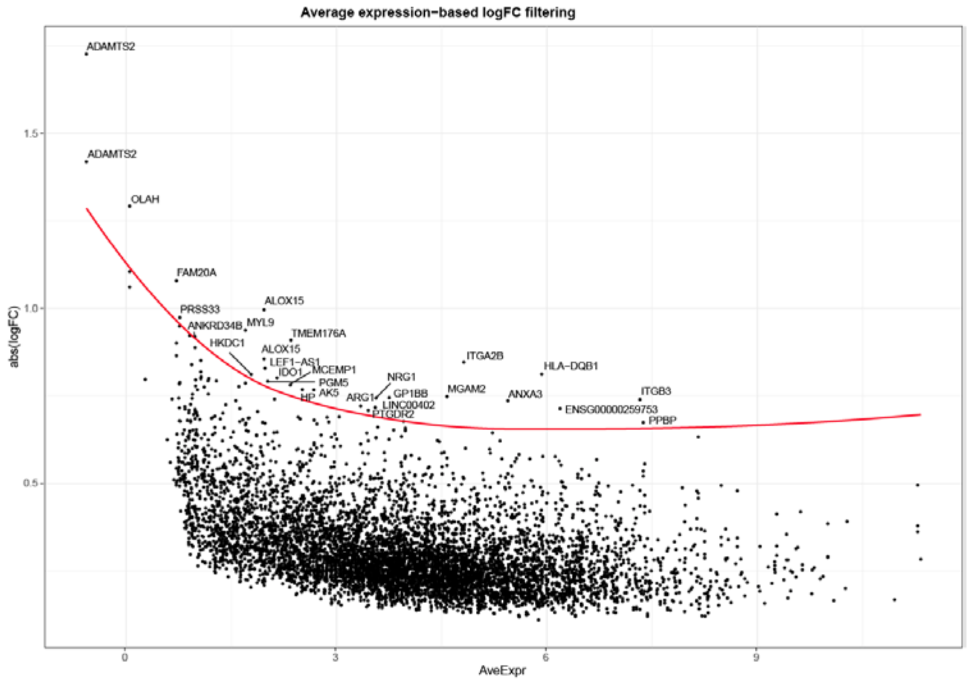

Supplementary Fig. S3. Candidate gene filtering approach. Our filtering approach used both average gene expression (AveExpr) and absolute $\log_2$ fold change (logFC) values. All significantly up- and downregulated differentially expressed (DE) genes identified in the case-control subcomparisons (all LC cases vs. FalsePos, non-small cell LC (NSCLC) cases vs. FalsePos and late-stage LC cases vs. FalsePos) were included, and a locally weighted smoothing (loess) regression curve with a stricter cut-off was fitted based on both AveExpr and logFC values. The stricter cut-off aimed in having up to 30 candidate genes to be tested in the prospective studies. Only genes with absolute logFC values above the gene expression-dependent loess curve were considered as potential LC candidates in our further analyses using prospective studies.

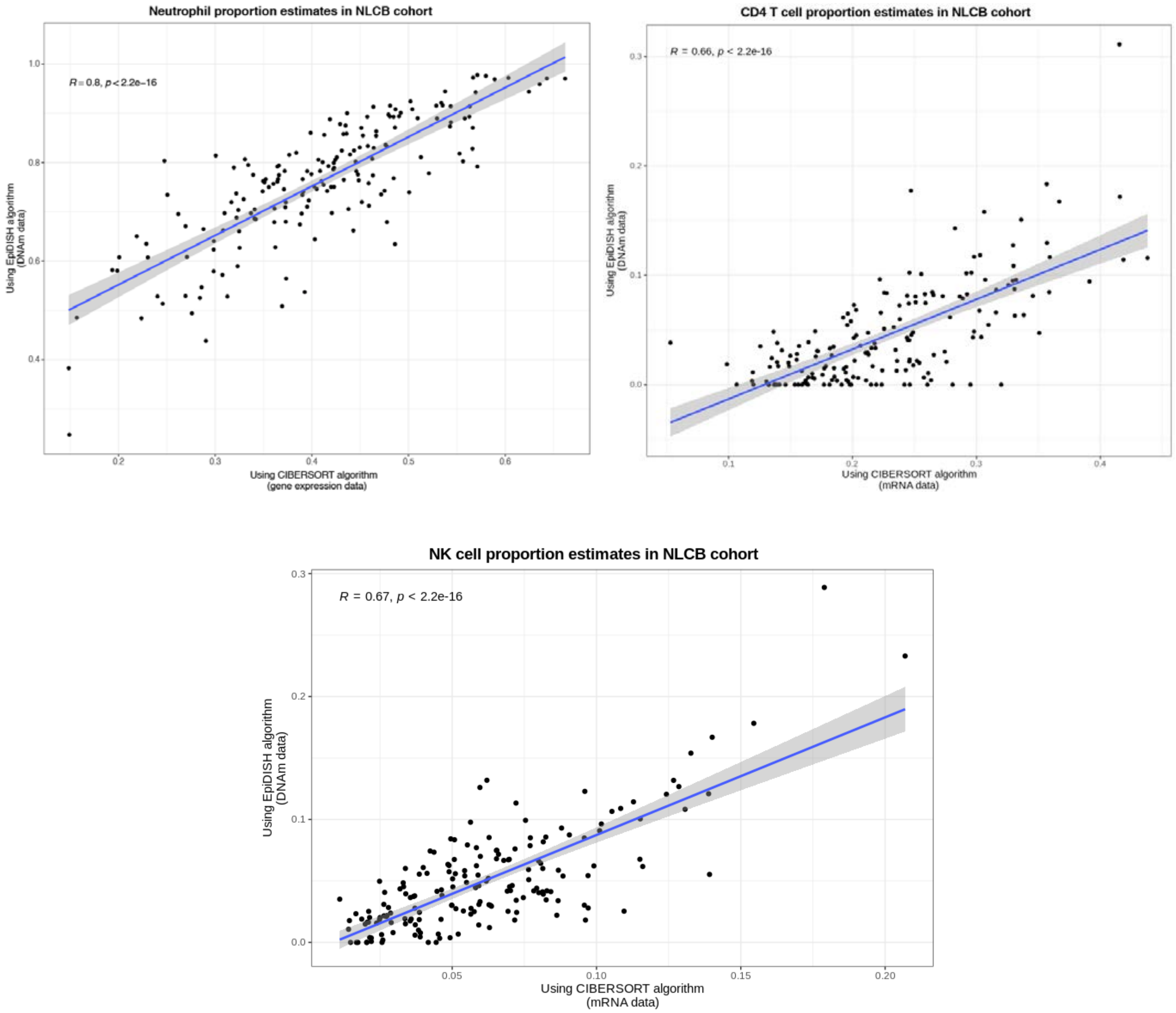

Supplementary Fig. S4. Blood cell type proportion estimates. Correlation of blood cell type proportions estimated by EpiDISH using DNA methylation (DNAm) data vs. by CIBERSORT using gene expression data for 178 specimens in the diagnostic study, with Spearman's rank correlation coefficient of 0.8 (R=0.8) for the neutrophil estimates, of 0.66 (R=0.66) for the CD4 T cell estimates and of 0.66 (R=0.67) for the NK cell estimates.

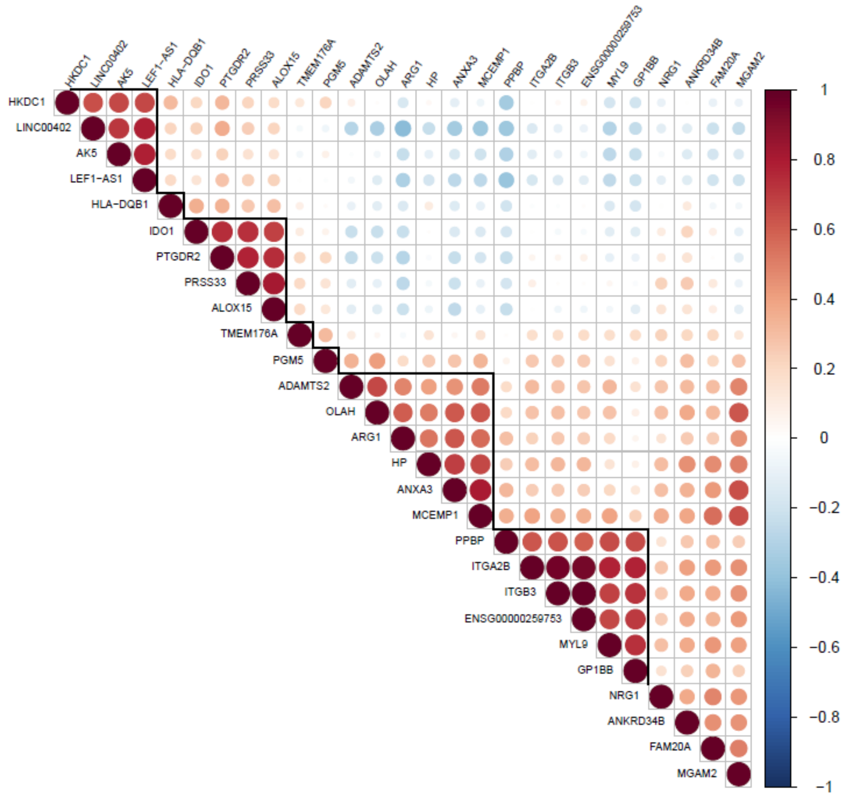

Supplementary Fig. S5. Correlation of candidate gene expression in the diagnostic study. Correlation matrix of $\log_2$ reads per million ($\log_2$RPM) values of the 27 unique candidates identified in the diagnostic study (Pearson correlation coefficients). Four highly correlated blocks are highlighted with black lines.

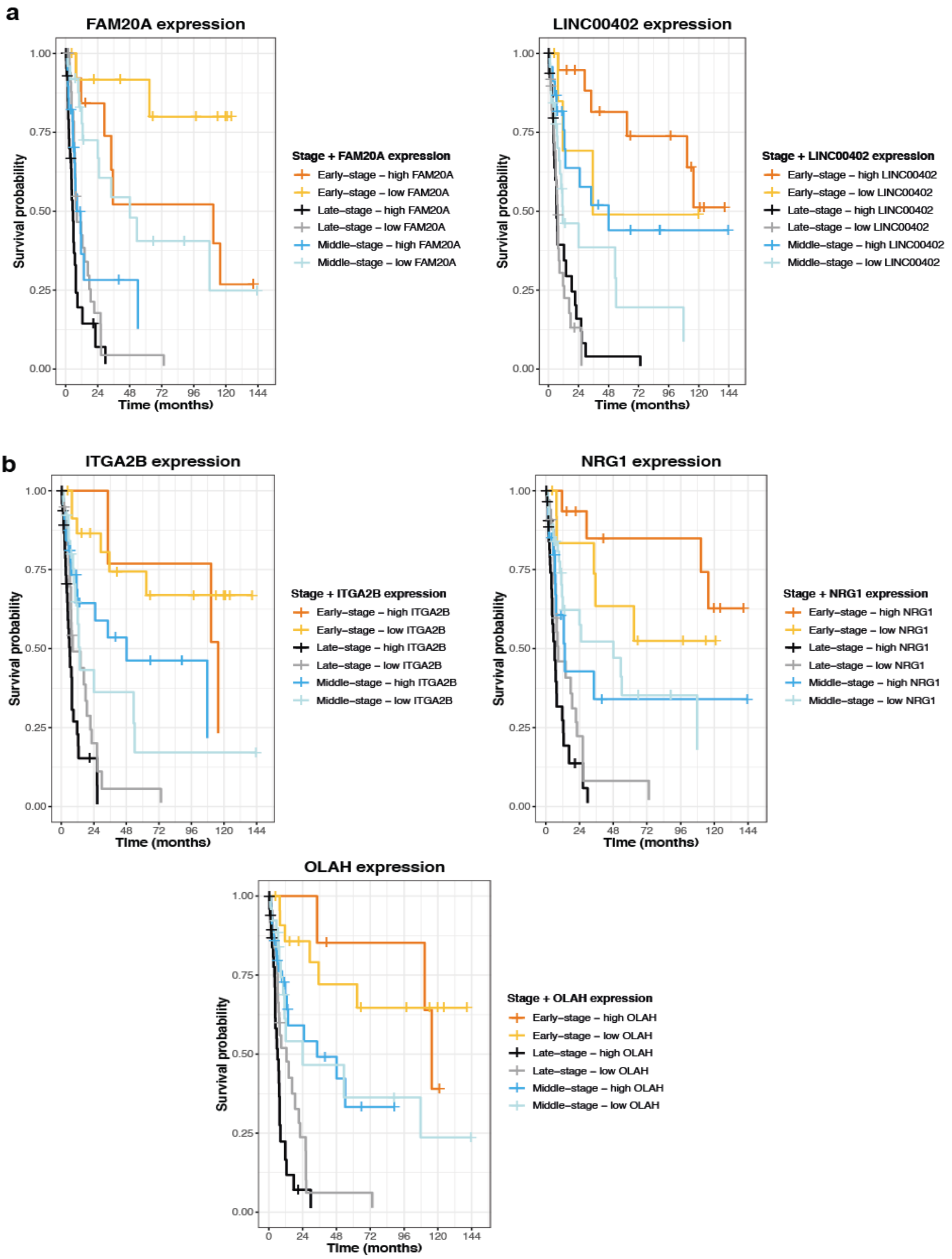

Supplementary Fig. S6. Survival curves in the diagnostic study. Expression of three candidate genes was significantly associated with survival in middle-stage LC cases: (a) *ANXA3* (FDR=4.08E-02), *FAM20A* (FDR=4.65E-03) and *LINC00402* (FDR=1.06E-02) and (b) six candidates in late-stage LC cases: *ANXA3* (FDR=9.18E-03), *ARG1* (FDR=3.15E-02), *HP* (FDR=6.93E-03), *ITGA2B* (FDR=2.87E-02), *NRG1* (FDR=4.11E-

02) and *OLAH* (FDR=2.32E-02). All FDR values are Benjamini-Hochberg adjusted p-values. Low or high gene expression was defined as having below or above median $\log_2$ reads per million ($\log_2$RPM) of this gene (indicated as ‘low’ or ‘high’, respectively). Survival curves for *ANXA3*, *ARG1* and *HP* are presented in the main text (Fig. 1g-i).

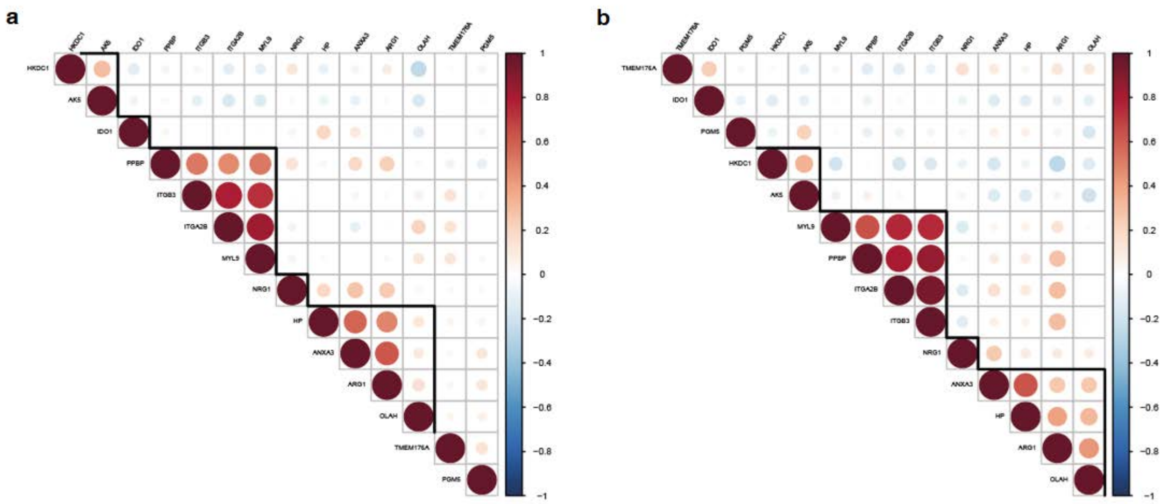

Supplementary Fig. S7. Correlation of candidate gene expression in the prospective studies. Correlation matrix of expression values (log_2RPM) of the 14 candidates evaluated in the prospective studies (a) NOWAC and (b) HUNT3 (Pearson correlation coefficients): adenylate kinase 5 (*AK5*), arginase 1 (*ARG1*), annexin A3 (*ANXA3*), haptoglobin (*HP*), hexokinase domain containing protein 1 (*HKDC1*), indoleamine 2,3-dioxygenase 1 (*IDO1*), integrin subunit alpha 2b (*ITGA2B*), integrin subunit beta 3 (*ITGB3*), myosin light chain 9 (*MYL9*), neuregulin 1 (*NRG1*), oleoyl-ACP hydrolase (*OLAH*), phosphoglucomutase 5 (*PGM5*), pro-platelet basic protein (*PPBP*) and transmembrane protein 176A (*TMEM176A*). Highly correlated genes identified in the diagnostic study (Fig. 1f) are highlighted with black lines.

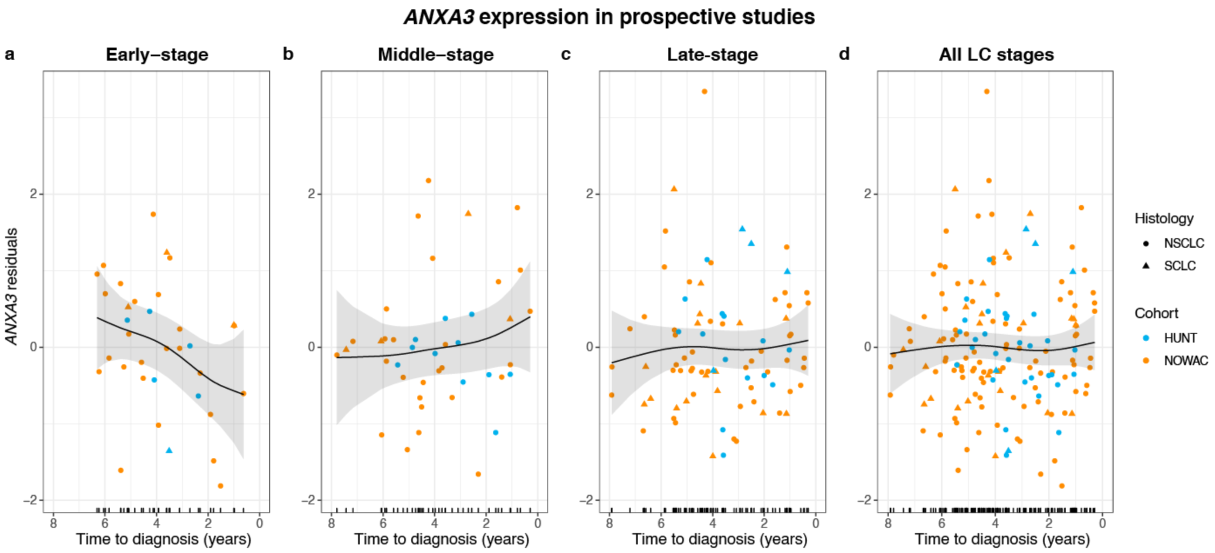

Supplementary Fig. S8. Trends of *ANXA3* expression in the prospective studies. (a) *ANXA3* expression in early-stage, (b) middle-stage (c) late-stage and (d) all LC stages in relation to time between specimen collection and LC diagnosis. The time trends are visualized using smoothing cubic regression spline curves from generalized additive models (gam, df=2). As input to these models, we used residuals from mixed models (lmer) that were adjusted for sex, age (scaled) and smoking variable (seven categories based on both smoking status [never/current/former] and pack-years) in addition to using study as random effect. Histological subtypes in these plots are visualized with different point shapes (NSCLC=non-small cell LC, SCLC=small cell LC) and studies are visualized with different point colours.

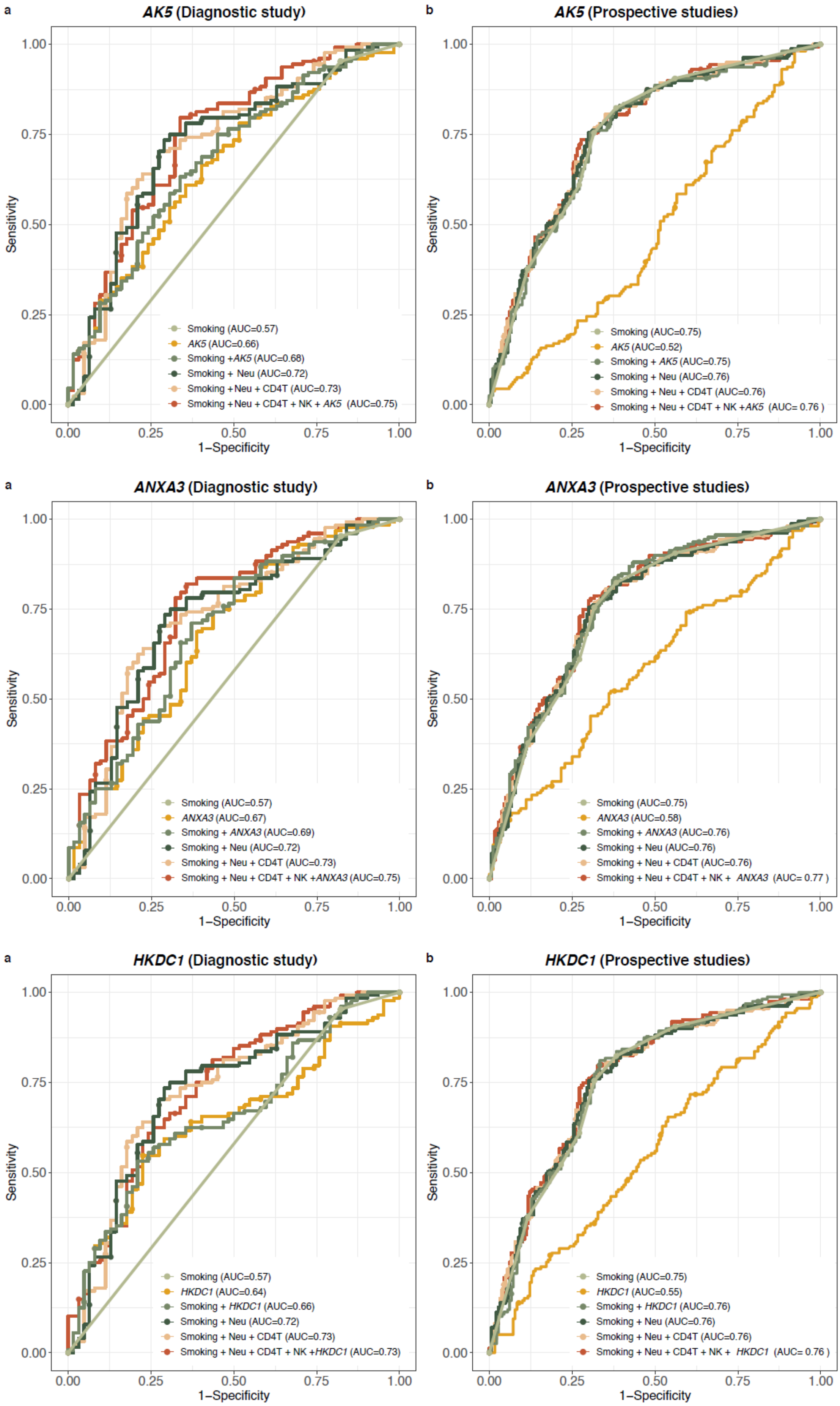

a
AK5 (Diagnostic study)
Sensitivity
1−Specificity
Smoking (AUC=0.57)
AK5 (AUC=0.66)
Smoking +AK5 (AUC=0.68)
Smoking + Neu (AUC=0.72)
Smoking +Neu + CD4T (AUC=0.73)
Smoking +Neu + CD4T + NK + AK5 (AUC=0.75)
b
AK5 (Prospective studies)
Sensitivity
1−Specificity
Smoking (AUC=0.75)
AK5 (AUC=0.52)
Smoking + AK5 (AUC=0.75)
Smoking + Neu (AUC=0.76)
Smoking + Neu + CD4T (AUC=0.76)
Smoking + Neu + CD4T + NK +AK5 (AUC= 0.76)
a
ANXA3 (Diagnostic study)
Sensitivity
1−Specificity
Smoking (AUC=0.57)
ANXA3 (AUC=0.67)
Smoking + ANXA3 (AUC=0.69)
Smoking + Neu (AUC=0.72)
Smoking + Neu + CD4T (AUC=0.73)
Smoking + Neu + CD4T + NK +ANXA3 (AUC=0.75)
b
ANXA3 (Prospective studies)
Sensitivity
1−Specificity
Smoking (AUC=0.75)
ANXA3 (AUC=0.58)
Smoking + ANXA3 (AUC=0.76)
Smoking + Neu (AUC=0.76)
Smoking + Neu + CD4T (AUC=0.76)
Smoking + Neu + CD4T + NK + ANXA3 (AUC= 0.77)
a
HKDC1 (Diagnostic study)
Sensitivity
1−Specificity
Smoking (AUC=0.57)
HKDC1 (AUC=0.64)
Smoking + HKDC1 (AUC=0.66)
Smoking + Neu (AUC=0.72)
Smoking + Neu + CD4T (AUC=0.73)
Smoking + Neu + CD4T + NK +HKDC1 (AUC=0.73)
b
HKDC1 (Prospective studies)
Sensitivity
1−Specificity
Smoking (AUC=0.75)
HKDC1 (AUC=0.55)
Smoking + HKDC1 (AUC=0.76)
Smoking + Neu (AUC=0.76)
Smoking + Neu + CD4T (AUC=0.76)
Smoking + Neu + CD4T + NK + HKDC1 (AUC= 0.76)

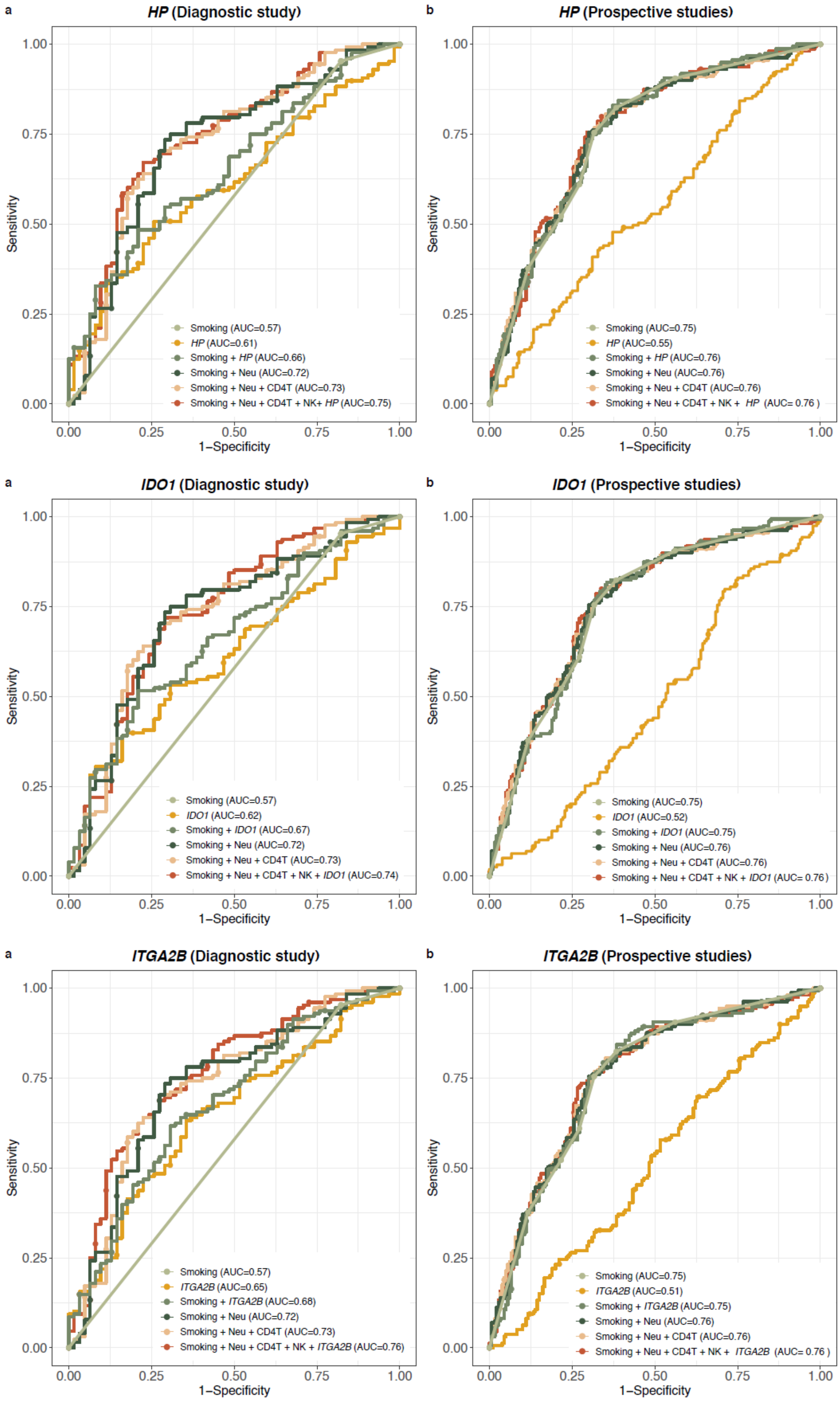

a
HP (Diagnostic study)
Sensitivity
1−Specificity
0.00
0.25
0.50
0.75
1.00
Smoking (AUC=0.57)
HP (AUC=0.61)
Smoking + HP (AUC=0.66)
Smoking + Neu (AUC=0.72)
Smoking + Neu + CD4T (AUC=0.73)
Smoking + Neu + CD4T + NK+ HP (AUC=0.75)
b
HP (Prospective studies)
Smoking (AUC=0.75)
HP (AUC=0.55)
Smoking + HP (AUC=0.76)
Smoking + Neu (AUC=0.76)
Smoking + Neu + CD4T (AUC=0.76)
Smoking + Neu + CD4T + NK + HP (AUC= 0.76)
a
IDO1 (Diagnostic study)
Smoking (AUC=0.57)
IDO1 (AUC=0.62)
Smoking + IDO1 (AUC=0.67)
Smoking + Neu (AUC=0.72)
Smoking + Neu + CD4T (AUC=0.73)
Smoking + Neu + CD4T + NK + IDO1 (AUC=0.74)
b
IDO1 (Prospective studies)
Smoking (AUC=0.75)
IDO1 (AUC=0.52)
Smoking + IDO1 (AUC=0.75)
Smoking + Neu (AUC=0.76)
Smoking + Neu + CD4T (AUC=0.76)
Smoking + Neu + CD4T + NK + IDO1 (AUC= 0.76)
a
ITGA2B (Diagnostic study)
Smoking (AUC=0.57)
ITGA2B (AUC=0.65)
Smoking + ITGA2B (AUC=0.68)
Smoking + Neu (AUC=0.72)
Smoking + Neu + CD4T (AUC=0.73)
Smoking + Neu + CD4T + NK + ITGA2B (AUC=0.76)
b
ITGA2B (Prospective studies)
Smoking (AUC=0.75)
ITGA2B (AUC=0.51)
Smoking + ITGA2B (AUC=0.75)
Smoking + Neu (AUC=0.76)
Smoking + Neu + CD4T (AUC=0.76)
Smoking + Neu + CD4T + NK + ITGA2B (AUC= 0.76)

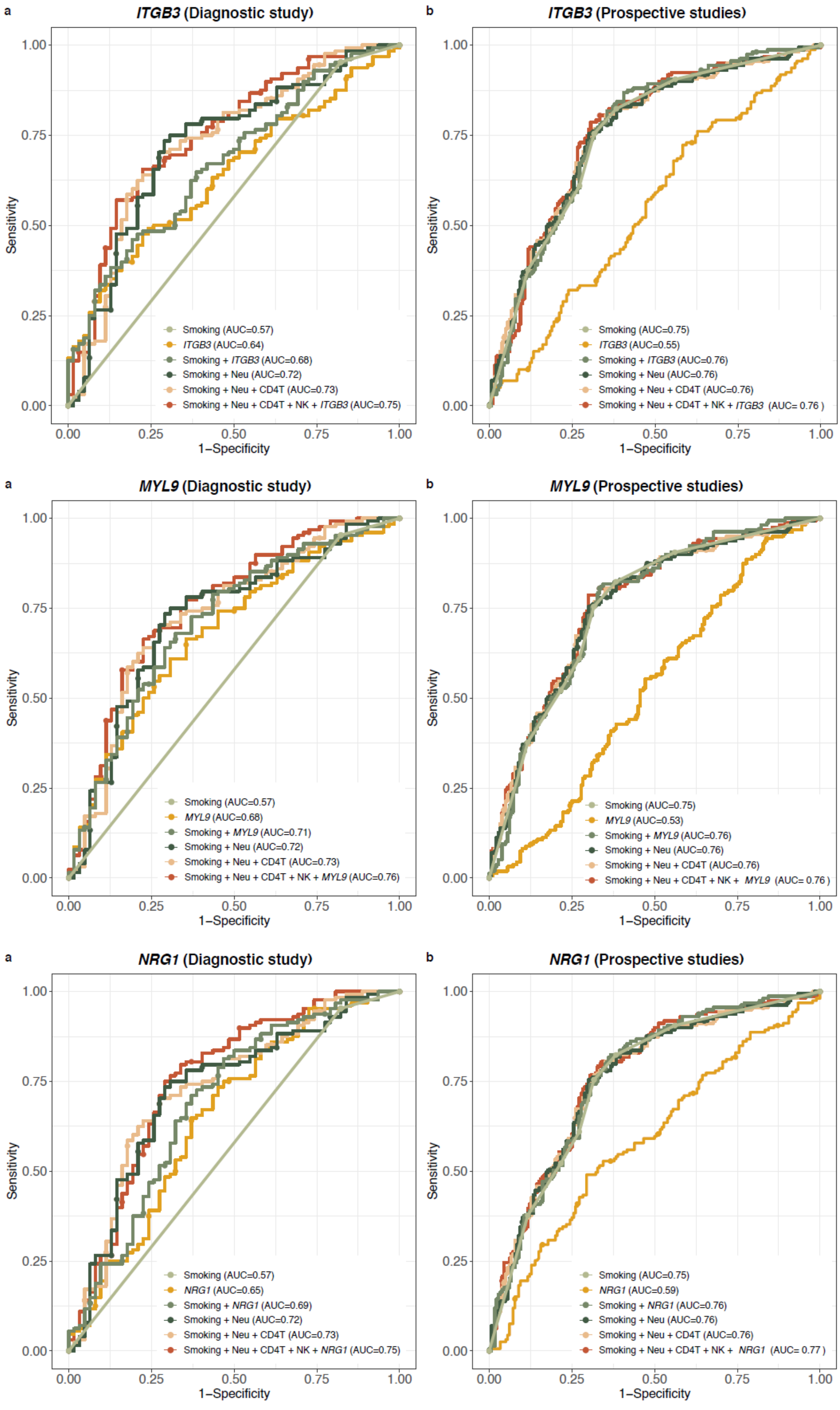
a
ITGB3 (Diagnostic study)
Sensitivity
1−Specificity
Smoking (AUC=0.57)
ITGB3 (AUC=0.64)
Smoking + ITGB3 (AUC=0.68)
Smoking + Neu (AUC=0.72)
Smoking + Neu + CD4T (AUC=0.73)
Smoking + Neu + CD4T + NK + ITGB3 (AUC=0.75)
b
ITGB3 (Prospective studies)
Sensitivity
1−Specificity
Smoking (AUC=0.75)
ITGB3 (AUC=0.55)
Smoking + ITGB3 (AUC=0.76)
Smoking + Neu (AUC=0.76)
Smoking + Neu + CD4T (AUC=0.76)
Smoking + Neu + CD4T + NK + ITGB3 (AUC= 0.76)
a
MYL9 (Diagnostic study)
Sensitivity
1−Specificity
Smoking (AUC=0.57)
MYL9 (AUC=0.68)
Smoking + MYL9 (AUC=0.71)
Smoking + Neu (AUC=0.72)
Smoking + Neu + CD4T (AUC=0.73)
Smoking + Neu + CD4T + NK + MYL9 (AUC=0.76)
b
MYL9 (Prospective studies)
Sensitivity
1−Specificity
Smoking (AUC=0.75)
MYL9 (AUC=0.53)
Smoking + MYL9 (AUC=0.76)
Smoking + Neu (AUC=0.76)
Smoking + Neu + CD4T (AUC=0.76)
Smoking + Neu + CD4T + NK + MYL9 (AUC= 0.76)
a
NRG1 (Diagnostic study)
Sensitivity
1−Specificity
Smoking (AUC=0.57)
NRG1 (AUC=0.65)
Smoking + NRG1 (AUC=0.69)
Smoking + Neu (AUC=0.72)
Smoking + Neu + CD4T (AUC=0.73)
Smoking + Neu + CD4T + NK + NRG1 (AUC=0.75)
b
NRG1 (Prospective studies)
Sensitivity
1−Specificity
Smoking (AUC=0.75)
NRG1 (AUC=0.59)
Smoking + NRG1 (AUC=0.76)
Smoking + Neu (AUC=0.76)
Smoking + Neu + CD4T (AUC=0.76)
Smoking + Neu + CD4T + NK + NRG1 (AUC= 0.77)

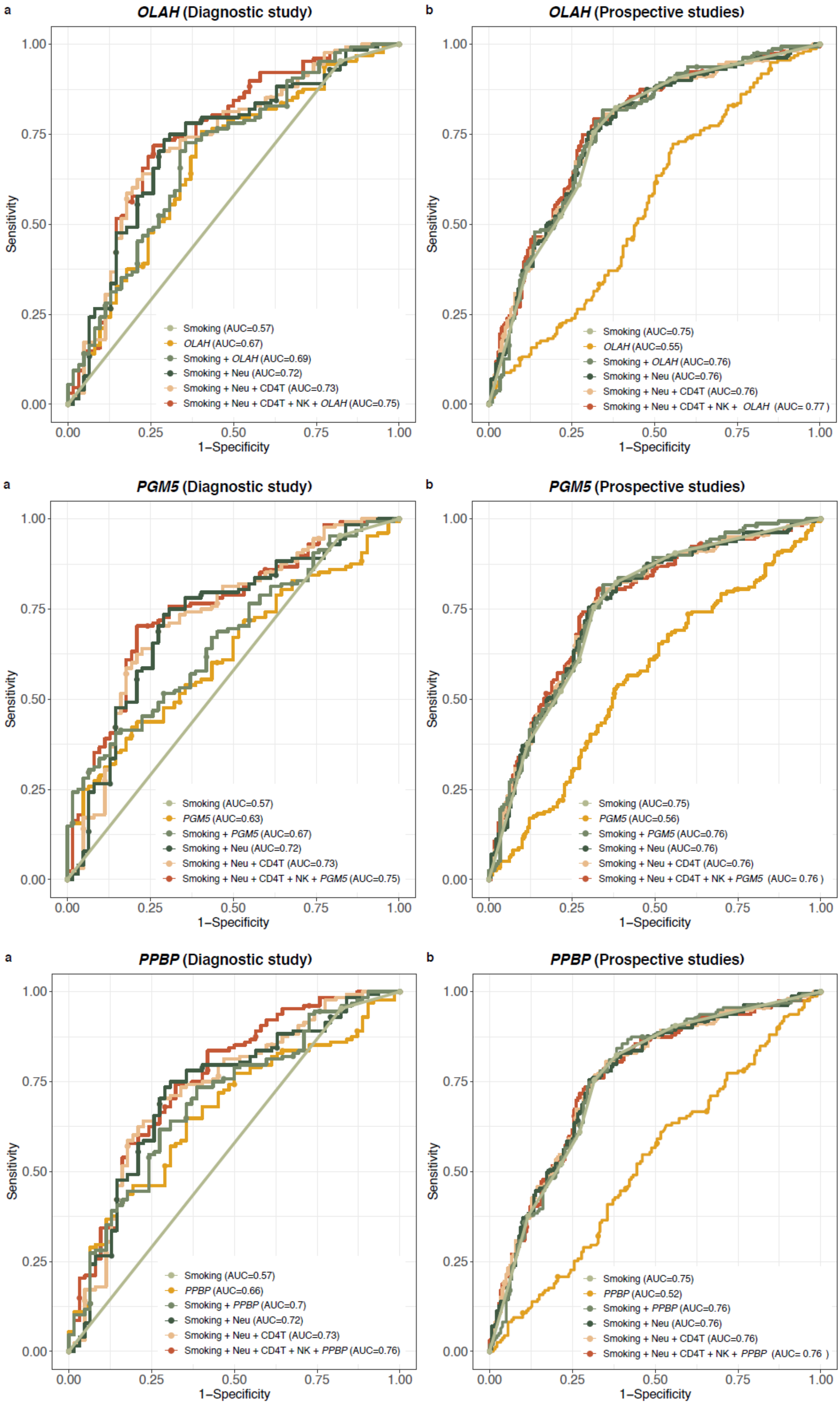
a
OLAH (Diagnostic study)
Sensitivity
1−Specificity
Smoking (AUC=0.57)
OLAH (AUC=0.67)
Smoking + OLAH (AUC=0.69)
Smoking + Neu (AUC=0.72)
Smoking + Neu + CD4T (AUC=0.73)
Smoking + Neu + CD4T + NK + OLAH (AUC=0.75)
b
OLAH (Prospective studies)
Sensitivity
1−Specificity
Smoking (AUC=0.75)
OLAH (AUC=0.55)
Smoking + OLAH (AUC=0.76)
Smoking + Neu (AUC=0.76)
Smoking + Neu + CD4T (AUC=0.76)
Smoking + Neu + CD4T + NK + OLAH (AUC= 0.77)
a
PGM5 (Diagnostic study)
Sensitivity
1−Specificity
Smoking (AUC=0.57)
PGM5 (AUC=0.63)
Smoking + PGM5 (AUC=0.67)
Smoking + Neu (AUC=0.72)
Smoking + Neu + CD4T (AUC=0.73)
Smoking + Neu + CD4T + NK + PGM5 (AUC=0.75)
b
PGM5 (Prospective studies)
Sensitivity
1−Specificity
Smoking (AUC=0.75)
PGM5 (AUC=0.56)
Smoking + PGM5 (AUC=0.76)
Smoking + Neu (AUC=0.76)
Smoking + Neu + CD4T (AUC=0.76)
Smoking + Neu + CD4T + NK + PGM5 (AUC= 0.76)
a
PPBP (Diagnostic study)
Sensitivity
1−Specificity
Smoking (AUC=0.57)
PPBP (AUC=0.66)
Smoking + PPBP (AUC=0.7)
Smoking + Neu (AUC=0.72)
Smoking + Neu + CD4T (AUC=0.73)
Smoking + Neu + CD4T + NK + PPBP (AUC=0.76)
b
PPBP (Prospective studies)
Sensitivity
1−Specificity
Smoking (AUC=0.75)
PPBP (AUC=0.52)
Smoking + PPBP (AUC=0.76)
Smoking + Neu (AUC=0.76)
Smoking + Neu + CD4T (AUC=0.76)
Smoking + Neu + CD4T + NK + PPBP (AUC= 0.76)

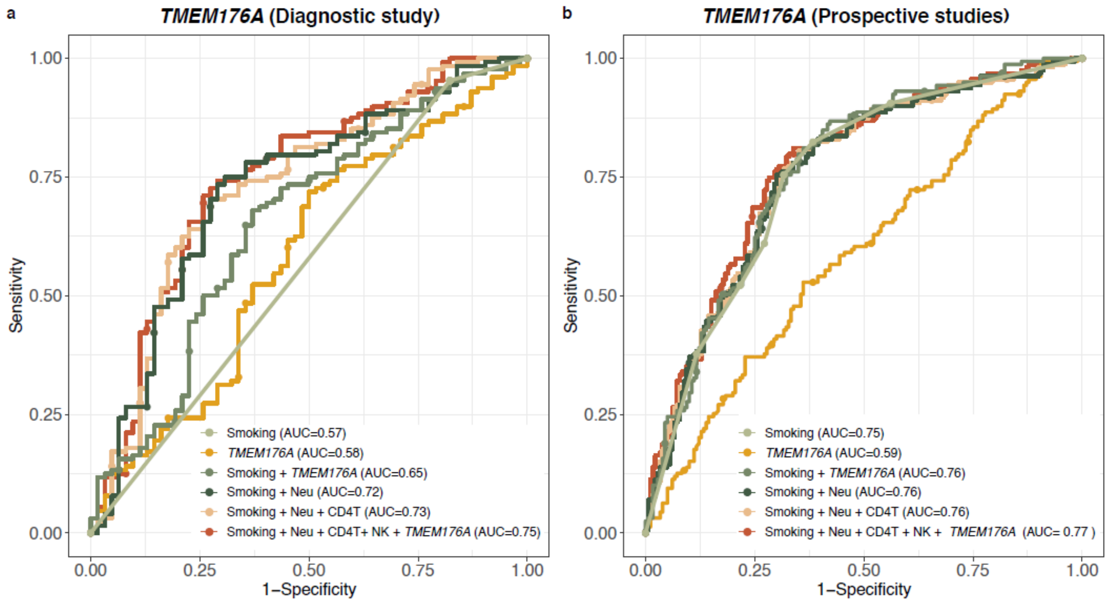

Supplementary Fig. S9. Receiver operating characteristic (ROC) curves in the diagnostic and prospective studies for all candidate genes. (a) LC discrimination based on 128 cases and 62 FalsePos in the diagnostic study (NLCB). (b) LC discrimination based on 163 cases and 184 controls in the prospective studies (NOWAC and HUNT3). Six separate ROC curves are visualized for models including (i) smoking status/ variable only ("Smoking"), (ii) candidate gene expression only (e.g. "*ANXA3*"), (iii) both the smoking status/ variable and candidate gene expression (e.g. "Smoking + *ANXA3*"), (iv) both the smoking status/ variable and blood neutrophil estimates ("Smoking + Neu"), (v) the smoking status/ variable, blood neutrophil and CD4 T cell estimates ("Smoking + Neu + CD4T") and (vi) the smoking status/ variable, blood neutrophil, CD4 T and NK cell estimates and candidate gene expression (e.g. "Smoking + Neu + CD4T + NK + *ANXA3*"). ROC curves of the smoking status/ variable, blood neutrophil, CD4 T cell and NK cell estimates ("Smoking + Neu + CD4T + NK") are not shown here, as they were very similar to the "Smoking + Neu + CD4T" ROC curves with almost identical AUC values. In the diagnostic study, smoking status was defined as never/ever smokers and in the prospective studies as smoking variable with seven categories combining smoking status (never/current/former) and pack-years (see Material and methods section in the main text for further description). ROC curves for all candidate genes excluding *ARG1* (Fig. 3) are shown here.

Supplementary Table S1. The definition of cancer stage categories based on medical records (diagnostic NLCB study) and information from the Cancer Registry of Norway (prospective NOWAC and HUNT3 studies).

Cancer stage	NLCB	NOWAC, HUNT3
	**Medical records – M in TNM status**	**Cancer Registry of Norway - metastasis categories***
Early-stage	Stage I-II	0
Middle-stage	Stage III	1, 5, 6
Late-stage	Stage IV	2, 3, 4, 7
Unknown	Unknown	9

** The coding of the categories in the registry was the following: 0 = No metastasis, 1 = Metastasis to regional lymph nodes, 2 = Metastasis to distant lymph nodes, 3 = Metastasis to organ in the same part of the body as the primary tumor, 4 = Metastasis to organ in another part of the body than the primary tumor, 5 = Microscopic growth into neighboring tissue, 6 = Macroscopic growth into neighboring tissue, 7 = Metastasis found, but uncertain where primary tumor is located, 8 = Microscopically infiltrating tumor, 9 = Unknown metastasis. None were coded 8*

Supplementary Table S4. Candidate genes identified in the diagnostic study. All 26 annotated candidate genes in the diagnostic study NLCB (N=190) that were identified from three comparisons with DE genes: all LC cases vs. FalsePos, non-small cell LC (NSCLC) cases vs. FalsePos and late-stage LC cases vs. FalsePos. Limma models were adjusted for age (scaled), sex, smoking status (never/ever) and technical variation.

**Gene**	**All LC cases vs. FalsePos**			**Late-stage LC cases vs. FalsePos**		**NSCLC cases vs. FalsePos**	
	AveExpr	logFC[1]	FDR value[2]	logFC[1]	FDR value[2]	logFC[1]	FDR value[2]
ADAMTS2	-0.41	1.45	**1.96E-02**	1.76	**6.13E-03**	1.17	**4.19E-02**
*AK5**	2.64	-0.60	**1.67E-02**	-0.78	**3.27E-03**	-0.51	**2.58E-02**
ALOX15	1.93	-1.05	**1.85E-02**	-0.89	**3.12E-02**	-0.82	**4.00E-02**
ANKRD34B	1.02	0.65	**3.68E-02**	0.95	**6.96E-03**	0.62	**4.68E-02**
*ANXA3**	5.46	0.52	**1.95E-02**	0.73	**3.27E-03**	0.52	**2.12E-02**
*ARG1**	3.35	0.61	**4.62E-02**	0.72	**3.03E-02**	0.54	7.88E-02
FAM20A	0.76	0.93	**2.88E-02**	1.10	**1.38E-02**	0.89	**3.55E-02**
GP1BB	3.80	0.54	**2.44E-02**	0.77	**3.90E-03**	0.53	**2.66E-02**
*HKDC1**	1.78	-0.55	**1.83E-02**	-0.80	**2.84E-03**	-0.57	**1.75E-02**
HLA-DQB1	5.94	-0.49	**1.47E-01**	-0.83	**2.77E-02**	-0.54	**1.21E-01**
*HP**	2.54	0.52	6.08E-02	0.77	**1.30E-02**	0.46	**1.03E-01**
*IDO1**	2.13	-0.81	**2.09E-02**	-0.64	5.68E-02	-0.65	**4.43E-02**
*ITGA2B**	4.86	0.53	**3.79E-02**	0.86	**4.69E-03**	0.50	5.48E-02
*ITGB3**	7.35	0.50	**3.82E-02**	0.76	**6.13E-03**	0.46	5.99E-02
LEF1-AS1	1.97	-0.68	**1.67E-02**	-0.84	**3.27E-03**	-0.65	**1.75E-02**
LINC00402	3.52	-0.47	**2.13E-02**	-0.71	**3.27E-03**	-0.45	**2.66E-02**
MCEMP1	2.34	0.57	**1.95E-02**	0.79	**3.27E-03**	0.52	**2.61E-02**
MGAM2	4.60	0.56	**1.67E-02**	0.75	**2.84E-03**	0.58	**1.75E-02**
*MYL9**	1.76	0.86	**1.67E-02**	0.99	**4.66E-03**	0.74	**2.42E-02**
*NRG1**	3.63	0.66	**2.58E-02**	0.76	**1.27E-02**	0.66	**2.66E-02**
*OLAH**	0.08	1.09	**1.95E-02**	1.27	**7.01E-03**	1.04	**2.42E-02**
*PGM5**	2.06	0.65	7.59E-02	0.86	**3.11E-02**	0.60	**1.04E-01**
*PPBP**	7.38	0.57	**1.67E-02**	0.70	**3.27E-03**	0.56	**1.75E-02**
PRSS33	0.72	-0.97	**2.28E-02**	-0.75	6.67E-02	-0.79	**4.76E-02**
PTGDR2	3.43	-0.67	**1.97E-02**	-0.55	**4.45E-02**	-0.59	**3.10E-02**
*TMEM176A**	2.47	0.72	6.29E-02	0.99	**2.08E-02**	0.73	6.42E-02

[1] *logFC = log_2 fold change*

[2] *False Discovery Rate (FDR)-adjusted p-values using Benjamini-Hochberg method*

** The 14 candidates that could be detected in the prospective studies datasets*

NSCLC=non-small cell LC

Supplementary Table S5. Candidate gene expression evaluated in the prospective studies. Case-control comparison of expression of our 14 candidate genes in the prospective studies (NOWAC and HUNT3 studies combined). Logistic mixed models (glmer) were adjusted for age (scaled), sex and smoking variable (seven categories) unless otherwise stated, with study as a random effect.

**Gene**	**NOWAC+HUNT3** *without smoking adjustment* [1]			**NOWAC+HUNT3** *with smoking adjustment* [2]			**NOWAC+HUNT3** *Late-stage LC cases only* [3]			**NOWAC+HUNT3** *NSCLC cases only* [4]		
	OR	95% CI	p-value	OR	95% CI	p-value	OR	95% CI	p-value	OR	95% CI	p-value
AK5	0.94	(0.70-1.25)	6.56E-01	1.07	(0.74-1.49)	7.00E-01	0.98	(0.66-1.47)	9.40E-01	1.12	(0.78-1.62)	5.33E-01
ANXA3	1.39	(1.09-1.77)	**8.89E-03**	1.32	(0.99-1.74)	5.47E-02	1.37	(0.97-1.94)	7.65E-02	1.27	(0.95-1.72)	1.11E-01
ARG1	1.53	(1.11-2.12)	**1.00E-02**	1.37	(0.96-2.00)	9.10E-02	1.44	(0.92-2.26)	1.08E-01	1.38	(0.94-2.01)	9.67E-02
HKDC1	1.11	(0.88-1.39)	3.82E-01	1.05	(0.80-1.38)	7.30E-01	1.00	(0.72-1.39)	9.85E-01	1.08	(0.81-1.44)	6.19E-01
HP	1.24	(1.00-1.54)	**4.59E-02**	1.14	(0.89-1.47)	2.84E-01	1.14	(0.84-1.55)	4.15E-01	1.14	(0.88-1.47)	3.35E-01
IDO1	0.93	(0.74-1.16)	5.08E-01	0.94	(0.71-1.22)	6.63E-01	0.93	(0.67-1.30)	6.82E-01	0.98	(0.73-1.30)	8.79E-01
ITGA2B	1.07	(0.80-1.45)	6.36E-01	0.97	(0.67-1.36)	8.53E-01	0.99	(0.64-1.52)	9.49E-01	1.04	(0.72-1.52)	8.22E-01
ITGB3	0.90	(0.74-1.10)	3.20E-01	0.88	(0.69-1.12)	3.16E-01	0.91	(0.67-1.22)	5.19E-01	0.89	(0.69-1.15)	3.69E-01
MYL9	1.10	(0.90-1.34)	3.42E-01	1.07	(0.85-1.35)	5.83E-01	1.03	(0.78-1.36)	8.28E-01	1.10	(0.86-1.39)	4.53E-01
NRG1	1.19	(0.96-1.46)	1.05E-01	1.12	(0.87-1.42)	3.59E-01	1.13	(0.83-1.53)	4.46E-01	1.09	(0.85-1.40)	4.97E-01
OLAH	1.11	(0.99-1.24)	6.66E-02	1.10	(0.98-1.25)	1.18E-01	1.07	(0.92-1.23)	3.93E-01	1.11	(0.98-1.27)	1.05E-01
PGM5	1.02	(0.89-1.18)	7.62E-01	1.08	(0.92-1.29)	3.51E-01	1.05	(0.86-1.30)	6.20E-01	1.12	(0.94-1.35)	2.04E-01
PPBP	0.90	(0.66-1.24)	5.19E-01	0.89	(0.61-1.28)	5.48E-01	0.90	(0.57-1.42)	6.42E-01	0.91	(0.62-1.34)	6.28E-01
TMEM176A	1.16	(1.04-1.30)	**1.06E-02**	1.19	(1.03-1.37)	**1.38E-02**	1.19	(1.00-1.43)	5.31E-02	1.20	(1.03-1.39)	**1.59E-02**

[1] *All LC cases without smoking status adjustment (161 cases, 184 controls)*

[2] *All LC cases with smoking status adjustment (161 cases, 184 controls)*

[3] *Late-stage LC cases only (85 cases, 184 controls)*

[4] *NSCLC cases only (132 cases, 184 controls)*

Supplementary Table S6. Candidate gene expression close to LC diagnosis. Case-control comparison of expression of our two candidate genes (*ANXA3*, *ARG1*) in prospective cases with < 2 years to LC diagnosis. Logistic mixed models (glmer) were adjusted for age (scaled), sex and smoking variable (seven categories) unless otherwise stated, with study as a random effect (glmer).

**Gene**	**NOWAC+HUNT3** *without smoking adjustment* [1] **<2 years to LC diagnosis**			**NOWAC+HUNT3** *with smoking adjustment* [2] **<2 years to LC diagnosis**			**NOWAC+HUNT3** *Late-stage LC cases only* [3] **<2 years to LC diagnosis**			**NOWAC+HUNT3** *NSCLC cases only* [4] **<2 years to LC diagnosis**		
	OR	95% CI	p-value	OR	95% CI	p-value	OR	95% CI	p-value	OR	95% CI	p-value
ANXA3	2.08	(1.32-3.30)	**1.69E-03**	2.22	(1.27-3.88)	**5.05E-03**	3.47	(1.53-7.86)	**2.85E-03**	2.05	(1.15-3.66)	**1.49E-02**
ARG1	2.91	(1.62-5.22)	**3.38E-04**	3.47	(1.69-7.10)	**6.70E-04**	5.00	(1.89-13.27)	**1.22E-03**	3.27	(1.58-6.78)	**1.41E-03**

[1] *All LC cases without smoking status adjustment (36 cases, 184 controls)*

[2] *All LC cases with smoking status adjustment (36 cases, 184 controls)*

[3] *Late-stage LC cases only (20 cases, 184 controls).*

[4] *NSCLC cases only (31 cases, 184 controls).*

Supplementary Table S7. Association of blood cell type estimates with LC. Blood cell type proportions estimated from DNA methylation data were assessed for associations with LC using logistic regression models (glm) adjusted for sex, age (scaled) and smoking status (never/ever) in the diagnostic study (NLCB), and adjusted for sex, age (scaled), smoking variable (seven categories) in the prospective studies (NOWAC and HUNT3).

**Blood cell type estimates**	**NLCB**			**NOWAC**			**HUNT3**		
	OR	95% CI	p-value	OR	95% CI	p-value	OR	95% CI	p-value
B	0.0001	8.73E-10-19.47	1.41E-01	0.0007	1.39E-08-38.37	1.93E-01	10.78	3.97E-05-2.92E+06	7.10E-01
NK	1.98E-06	3.81E-10-0.01	**2.63E-03**	1.03	0.004-254.30	9.92E-01	1.04	3.91E-13-2.77	6.80E-02
CD4T	9.99E-05	7.34E-08-0.14	**1.23E-02**	0.08	0.001-5.12	2.36E-01	0.34	3.90E-05-2.97E+03	8.16E-01
CD8T	0.004	1.53E-05-1.23	5.91E-02	0.13	0.0006-26.14	4.49E-01	1.39	1.13E-11-1.72	**2.42E-02**
Monocytes	0.0002	8.83E-11-476.1	2.56E-01	0.007	1.61E-07-278.77	3.56E-01	452.25	5.38E-10-3.80E+14	6.63E-01
Neutrophils	97.92	6.18-1552.43	**1.15E-03**	6.14	0.64-59.14	1.16E-01	45.35	0.37-5.48635E+03	1.19E-01
Eosinophils	29.82	8.12E-14-1.09E+16	8.43E-01	5.25E-10	1.32E-23-2.08E+04	1.81E-01	1.08	2.32E-162.5E+149	9.40E-01

Supplementary Table S8. Association of blood cell type estimates with LC. Blood cell type proportions estimated from gene expression data were assessed for associations with LC using logistic regression models (glm) adjusted for sex, age (scaled) and smoking status (never/ever) in the diagnostic study (NLCB), and adjusted for sex, age (scaled), smoking variable (seven categories) in the HUNT3 study and for age (scaled) and smoking variable (seven categories) in the NOWAC study (all females).

**Blood cell type estimates**	**NLCB**			**NOWAC**			**HUNT3**		
	OR	95% CI	p-value	OR	95% CI	p-value	OR	95% CI	p-value
B cells naive	0.027	1.51E-10-5.05E+06	7.12E-01	1.65E-31	1.06E-64-2.58E+02	6.91E-02	20.23	2.33E-07-1.75E+09	7.47E-01
B cells memory	2.64E-05	2.62E-55-2.66E+45	8.58E-01	1.73E+08	0.01-2.72E+18	1.13E-01	3.80E+22	0.009-1.68E+47	7.25E-02
Plasma cells	6.83E+25	5.39E-13-8.65E+63	1.84E-01	1.74E-19	1.73E-82-1.76E+44	5.60E-01	1.59E-18	8.37E-58-3.00E+21	3.74E-01
CD8 T cells	0.0056	4.20E-07-7.47E+01	2.85E-01	0.32	0.01-9.21	5.09E-01	0.001	7.93E-11-1.82E+04	4.25E-01
CD4 T cells naive	4.64E-05	2.69E-08-0.08	**8.67E-03**	0.13	0.0003-54.3	9.88E-01	0.0003	2.44E-09-48.71	1.88E-01
CD4 T cells memory resting	0.00017	1.63E-07-0.17	**1.37E-02**	0	0-Inf	8.75E-01	3.15E-09	1.57E-17-0.63	**4.47E-02**
CD4 T cells memory activated	2.27E-07	1.19E-23-4.32E+09	4.24E-01	0.22	1.70E-09-2.90E+07	4.49E-01	0.0003	2.20E-24-5.00E+16	7.35E-01
CD4 T cells follicular helper	1.72	1.22-2.43	**2.05E-03**	5.11E+22	9.08E-37-2.88E+81	2.93E-01	1.98	1.04-3.77	**3.78E-02**
Regulatory T cells (Tregs)	1.72	1.22-2.43	**2.05E-03**	0.016	7.82E-06-34.75	6.20E-01	1.98	1.04-3.77	**3.78E-02**
T cells gamma delta	1.93E+30	2.20E-05-1.69E+65	8.94E-02	7.12	0.0031-1.65E+04	7.44E-01	3.02E+21	1.85E-140-4.94E+182	7.94E-01
NK cells resting	4.69E-08	2.47E-12-0.00089	**7.88E-04**	2.96	0.0044-1.997E+03	7.29E-01	1.03E-07	7.70E-18-1.38E+03	1.76E-01
NK cells activated	Inf	0-Inf	9.91E-01	0.24	8.11E-05-7.28E+02	4.26E-01	1.80E+34	8.13E-59-4.00E+126	4.67E-01
Monocytes	1.80	0.02-1.58E+02	7.97E-01	0.12	0.0007-21.55	9.12E-01	5.53E+04	0.23-1.34E+10	8.44E-02
Macrophages M0	1.55E+04	2.84E-15-8.44E+22	6.61E-01	0.37	9.66-1.44E+07	7.55E-02	2.01E+04	2.63E-26-1.54E+34	7.78E-01
Macrophages M1	1.72	1.22-2.43	**2.05E-03**	9.46E+70	5.04E-08-1.76E+149	6.32E-01	Inf	0-Inf	9.92E-01
Macrophages M2	Inf	0-Inf	9.88E-01	1.58E+04	1.06E-13-2.36E+21	1.57E-01	1.98	1.04-3.77	**3.78E-02**
Dendritic cells resting	0	0-Inf	9.86E-01	1.12E+58	5.11E-23-2.46E+138	8.85E-01	0	0-Inf	6.80E-01
Dendritic cells activated	1.68E+17	1.30E-29-2.16E+63	4.64E-01	66.8	1.26E-23-3.55E+26	3.91E-01	1.12E-16	1.04-1.21E+07	1.75E-01
Mast cells resting	8.26E+16	1.51-4.51E+33	**4.76E-02**	1.59E-07	4.65E-23-5.45E+08	8.02E-01	1.76E+38	5.21E+07-5.95E+68	**1.41E-02**
Mast cells activated	1.72	1.22-2.43	**2.05E-03**	2.58E+04	9.26E-31-7.17E+38	9.43E-01	1.98	1.04-3.77	**3.78E-02**
Eosinophils	1.71E+33	1.32E-112-2.22E+178	6.54E-01	1.09E+13	0-Inf	9.43E-01	8.55E-89	3.16E-181-2.31E+04	6.18E-02
Neutrophils	1.92E+02	6.99-5.28E+03	**1.87E-03**	33.85	0.72-1.59E+03	7.30E-02	46.69	0.03-6.79E+04	3.01E-01
